# Absorption and Emission Spectroscopic Investigation of the Thermal Dynamics of the Archaerhodopsin 3 Based Fluorescent Voltage Sensor QuasAr1

**DOI:** 10.3390/ijms20174086

**Published:** 2019-08-21

**Authors:** Alfons Penzkofer, Arita Silapetere, Peter Hegemann

**Affiliations:** 1 Fakultät für Physik, Universität Regensburg, Universitätsstraße 31, D-93053 Regensburg, Germany; 2 Experimentelle Biophysik, Institut für Biologie, Humboldt Universität zu Berlin, Invalidenstraße 42, D-10115 Berlin, Germany

**Keywords:** QuasArs, Archaerhodopsin 3, genetically encoded voltage sensors (GEVIs), absorption spectroscopic characterization, fluorescence spectroscopic characterization, apparent protein melting temperature, thermal stability, thermal isomerization, thermal deprotonation

## Abstract

QuasAr1 is a fluorescent voltage sensor derived from Archaerhodopsin 3 (Arch) of *Halorubrum sodomense* by directed evolution. Here we report absorption and emission spectroscopic studies of QuasAr1 in Tris buffer at pH 8. Absorption cross-section spectra, fluorescence quantum distributions, fluorescence quantum yields, and fluorescence excitation spectra were determined. The thermal stability of QuasAr1 was studied by long-time attenuation coefficient measurements at room temperature (23 ± 2 °C) and at 2.5 ± 0.5 °C. The apparent melting temperature was determined by stepwise sample heating up and cooling down (obtained apparent melting temperature: 65 ± 3 °C). In the protein melting process the originally present protonated retinal Schiff base (PRSB) with absorption maximum at 580 nm converted to de-protonated retinal Schiff base (RSB) with absorption maximum at 380 nm. Long-time storage of QuasAr1 at temperatures around 2.5 °C and around 23 °C caused gradual protonated retinal Schiff base isomer changes to other isomer conformations, de-protonation to retinal Schiff base isomers, and apoprotein structure changes showing up in ultraviolet absorption increase. Reaction coordinate schemes are presented for the thermal protonated retinal Schiff base isomerizations and deprotonations in parallel with the dynamic apoprotein restructurings.

## 1. Introduction

Changes in electrical potential across the plasma membrane of neurons are important for intercellular and intracellular signal transmission [1]. Classical electrophysiology techniques involve placing electrodes into biological tissue allowing to record membrane currents [2,3]. Optical recordings of membrane potential from cells, especially neurons, with fluorescent voltage sensitive dyes [4,5,6,7,8], genetically encoded calcium indicators (GECIs) [9,10,11,12,13,14], and with fluorescent genetically encoded voltage indicators (GEVIs) [15,16,17,18,19,20,21,22,23,24,25,26,27,28] is an active field of research. Two major groups of GEVIs are i) integral membrane voltage sensing domains (VSDs) composed of four trans-membrane helices fused to fluorescent proteins [15,17,18,19,20,25,26,27,29,30,31,32,33,34] and ii) microbial rhodopsins composed of 7 trans-membrane α-helices with covalently bound retinal isomers [14,16,25,26,27,35,36,37,38]. Generally microbial rhodopsins exhibit low fluorescence quantum yield in the range of ϕ_F_ = 2 × 10^−4^ to 10^−5^ [39,40,41] which is too low for any cellular application. Directed evolution approach yielded modified microbial rhodopsins with increased fluorescence quantum yield, and few of them exhibited change of the fluorescence intensity depending on the membrane voltage [14,15,25,26,27,35,36,37,38,42,43,44,45,46,47,48]. In rhodopsin-fluorescent protein GEVIs a microbial rhodopsin is fused with a highly fluorescent protein and the emission of the fused fluorescent protein changes upon membrane voltage changes [16,28,49,50].

Archaerhodopsin 3 (Arch) from *Halorubrum sodomense* with a single residue mutation D95N showed potential as a GEVI [36] and initiated the development of the Arch variants QuasAr1 and QuasAr2 (named according to ‘Quality superior to Arch’) [42]. QuasArs have improved fluorescence intensity and membrane voltage sensitivity [22,42,45]. The fluorescence quantum yield of wild-type Arch was reported to be ϕ_F_ = 9 × 10^−4^ [36], of the mutant Arch D95N it was ϕ_F_ = 4 × 10^−4^ [36,42], while for QuasAr1 it increased to ϕ_F_ = 8 × 10^−3^ [42], and for QuasAr2 it was found to be ϕ_F_ = 4 × 10^−3^ [42]. The lower fluorescence quantum yield of QuasAr2 compared to QuasAr1 is compensated by its higher voltage sensitivity [42]. QuasAr1 differs from the wild-type Arch by 5 mutations, namely P60S, T80S, D95H, D106H, and F161V [42]. The amino acid sequence of QuasAr1 is shown in Figure S1 of the Supplementary Materials (Section S1). Some structural formulae of retinal cofactors of rhodopsins are found in [51] and are shown in Figure S2 of the Supplementary Materials (Section S2). QuasAr2 differs from QuasAr1 by the counter ion mutation H95Q.

Here a detailed study is presented of the absorption and emission spectroscopic properties and the thermal dynamics of QuasAr1 in pH 8 Tris buffer. Aliquots of 30 μL were used in the studies. They were stored at −80 °C and thawed before usage. The absorption cross-section spectrum, excitation wavelength dependent fluorescence emission quantum distributions and quantum yields, and emission wavelength dependent fluorescence excitation spectra of purified QuasAr1 were determined. The thermal stability of QuasAr1 was studied by long-time spectroscopic studies at room temperature (21–25 °C) and refrigerator temperature of 2.5 ± 0.5 °C. The apparent melting temperature was determined by stepwise sample heating up and cooling down. The temperature and time dependent retinal chromophore and opsin protein changes are discussed.

## 2. Results

### 2.1. Absorption and Emission Behavior of Fresh Thawed QuasAr1 Samples

**Figure 1:**
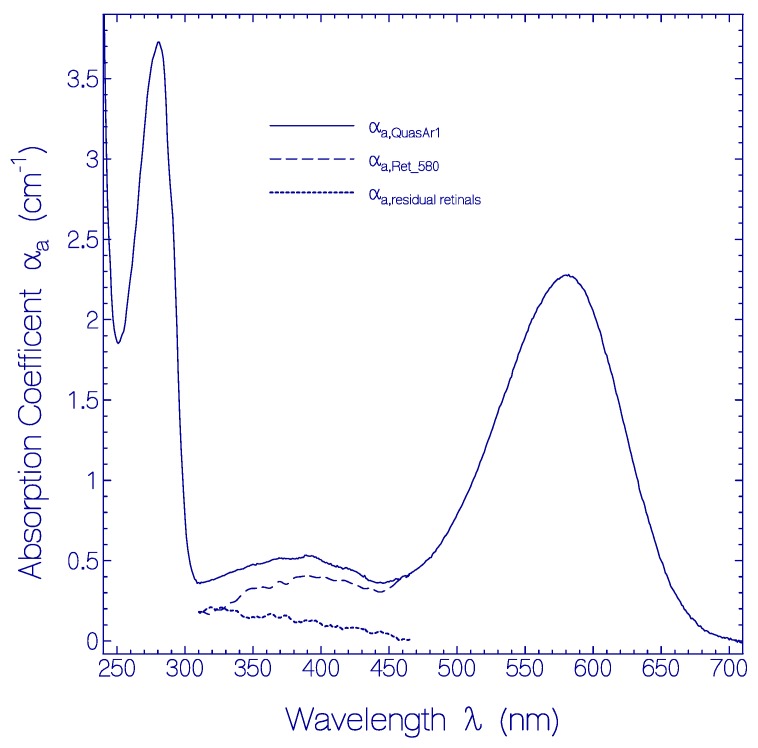
Absorption coefficient spectrum of a fresh thawed QuasAr1 sample in pH 8 Tris buffer. Solid curve: measured absorption coefficient spectrum α_a,QuasAr1_(λ). Dashed curve: absorption coefficient spetrum α_a,Ret_580_(λ) of PRSB Ret_580. Dotted curve: absorption coefficient spectrum of residual retinal components α_a,residual retinals_(λ) = α_a,QuasAr1_(λ) − α_a,Ret_580_(λ).

The absorption coefficient spectrum α_a_(λ) of a fresh thawed QuasAr1 sample was measured after centrifugation with 4400 rpm for 30 min at 4 °C (Centrifuge 5702 R, Eppendorf AG, Hamburg, Germany). It is displayed by the solid curve in Figure 1. The main absorption band with maximum at wavelength λ ≈ 580 nm is attributed to the singlet S_0_–S_1_ transition of protonated retinal Schiff base (PRSB) and named Ret_580. The absorption in the range from 310 nm to 465 nm is thought to be comprised of singlet S_0_-S_n_ (n ≥ 2) transitions of Ret_580 (dashed curve α_a,Ret_580_(λ) in Figure 1, for its determination see section S3 of the Supplementary Materials) and singlet ground-state–excited-state transitions of residual retinal components (dotted curve α_a,residual retinals_(λ) = α_a,QuasAr1_(λ) − α_a,Ret_580_(λ) in Figure 1). The short-wavelength absorption band peaking at λ = 280 nm is determined by apoprotein absorp tion (Trp, Tyr, Phe) and some retinal contribution.

The absorption cross-section spectrum of Ret_580, i.e., $\sigma_{a}(\lambda )=\alpha_{a,\mathrm{Ret}\_580}(\lambda )/N_{\mathrm{Ret}\_580}$ where *N*_Ret_580_ is the number density of Ret_580 chromophores in QuasAr1, is determined in section S3 of the Supplementary Materials (Figure S3).

**Figure 2:**
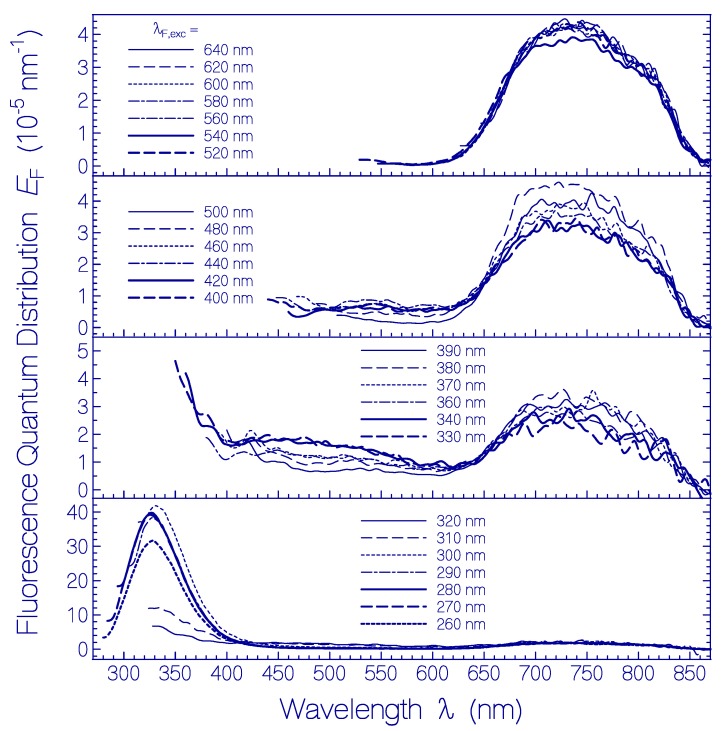
Fluorescence emission quantum distributions *E*_F_(λ) of fresh thawed QuasAr1 in pH 8 Tris buffer. The fluorescence excitation wavelengths λ_F,exc_ are indicated in the sub-figures.

**Figure 3:**
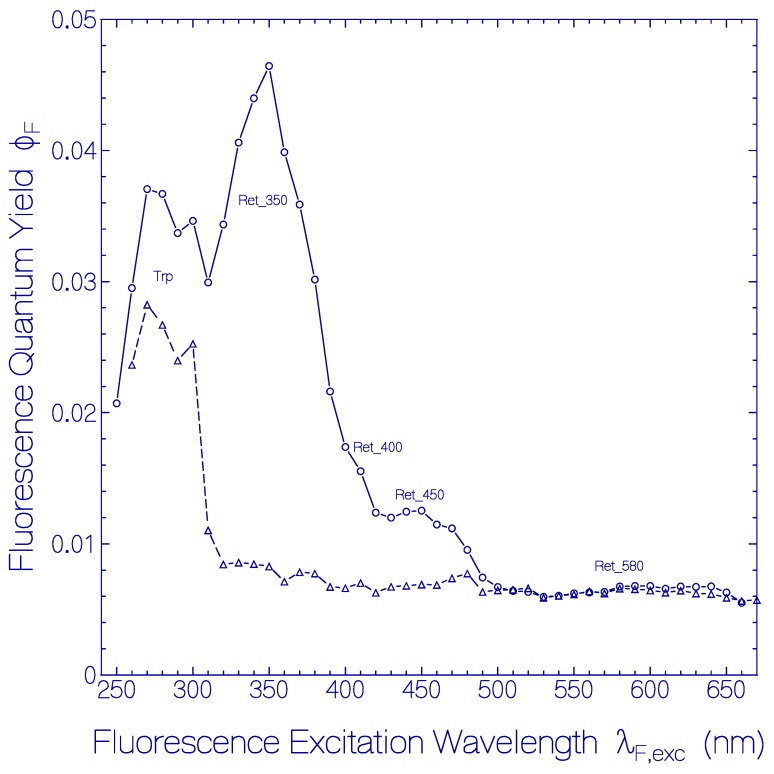
Dependence of the total fluorescence quantum yield ϕ_F_ on the fluorescence excitation wavelength λ_F,exc_ for QuasAr1 in pH 8 Tris buffer. The dashed line connected triangles belong to a fresh thawed sample. The line-connected circles belong to a sample stored at 2.5 ± 0.5 °C in the dark over a period of 80 days.

Fluorescence emission quantum distributions *E*_F_(λ) of a fresh thawed QuasAr1 sample in pH 8 Tris buffer for fluorescence excitation wavelengths λ_F,exc_ in the range from 260 nm to 640 nm are shown in Figure 2 and the corresponding fluorescence quantum yields are included in Figure 3 (see below). For λ_F,exc_ > 500 nm only S_1_–S_0_ emission from Ret_580 is observed. The wavelength position of peak fluorescence emission is at λ_F,max_ ≈ 740 nm. The full spectral half-width of this emission is $\delta {\tilde{\nu }}_{F,\mathrm{Ret}\_580}$ ≈ 2900 cm^−1^. The Stokes shift is $\delta {\tilde{\nu }}_{Stokes}=\lambda_{a,\max}^{-1}-\lambda_{F,\max}^{-1}$ ≈ 3730 cm^−1^. The strong Stokes shift and broad spectral width of the Ret_580 emission spectra indicate fluorescence emission along the S_1_ excited state photo-isomerization path. Below it will be shown that Ret_580 is composed dominantly of two protonated retinal Schiff base isomers (Ret_580_I_ and Ret_580_II_) in different apoprotein conformations (Apoprotein_I_ and Apoprotein_II_) which contribute to the broad fluorescence emission. In the Supplementary Materials (Table S1) wavelength positions of absorption maxima and fluorescence maxima together with Stokes shifts are listed for several rhodopsins (range of Stokes shifts from $\delta {\tilde{\nu }}_{Stokes}$ ≈ 1750 cm^−1^ for histidine kinase rhodopsin 1 from *Chlamydomonas reinhardtii* to $\delta {\tilde{\nu }}_{Stokes}$ ≈ 4900 cm^−1^ for proteorhodopsin from uncultivated marine γ-proteobacteria).

Fluorescence excitation in the wavelength range from 400 nm to 480 nm indicates an additional fluorescence emission band around λ_F,max_ ≈ 550 nm. It is thought to be caused by a small amount of a protonated retinal Schiff base isomer (named Ret_450, see below) absorbing in this range ($\delta {\tilde{\nu }}_{Stokes}$ ≈ 4000 cm^−1^). Fluorescence excitation in the range from 330 nm to 390 nm resulted in a broad fluorescence emission band around λ_F,max_ ≈ 470 nm. It is attributed to fluorescence emission of deprotonated retinal Schiff base isomer components. For fluorescence excitation in the wavelength region from 260 nm to 320 nm the fluorescence emission is dominated by Trp emission of the QuasAr1 apoprotein. The fluorescence emission maximum occurs at λ_F,max_ = 328 nm ($\delta {\tilde{\nu }}_{F,\mathrm{Trp}}$ ≈ 5600 cm^−1^, $\delta {\tilde{\nu }}_{Stokes}$ ≈ 5200 cm^−1^). For all excitation wavelengths, the Ret_580 fluorescence emission band around λ_F,max_ = 740 nm is present since the Ret_580 absorption extends over the whole applied fluorescence excitation wavelength region due to S_0_-S_n_ transitions (*n* ≥ 1) with fast S_n_–S_1_ nonradiative relaxation for *n* ≥ 2 and S_1_–S_0_ radiative emission. Additionally Förster-type energy transfer [52,53] occurs from Tyr and Trp to Ret_580 in the case of Tyr and Trp photo-excitation.

The dependence of the total fluorescence quantum yield $\phi_{F}=\int_{em}E_{F}(\lambda )d\lambda$ (the integration runs over the whole fluorescence emission wavelength region) of fresh thawed QuasAr1 on the fluorescence excitation wavelength λ_F,exc_ is depicted by the dashed curve connected triangles in Figure 3. The fluorescence quantum yield is ϕ_F_ = (6.5 ± 0.5) × 10^−3^ for excitation in the wavelength region of S_0_–S_1_ absorption of Ret_580 (λ_F,exc_ ≥ 490 nm). In the range of 380 nm ≤ λ_F,exc_ ≤ 480 nm it is ϕ_F_ = (7 ± 0.5) × 10^−3^, and in the range 320 nm ≤ λ_F,exc_ ≤ 380 nm it is ϕ_F_ = (8 ± 0.5) × 10^−3^ indicating a somewhat increased fluorescence efficiency of the additionally present retinal species besides Ret_580. In the range of 260 nm ≤ λ_F,exc_ ≤ 310 nm the fluorescence emission is dominated by apoprotein Trp emission. There the fluorescence quantum yield increased to ϕ_F_ = 0.026 ± 0.002. The fluorescence emission of photo-excited Tyr is quenched by Förster-type energy transfer [52,53] to Trp (see supplementary material to [54]). The Trp fluorescence is reduced by Förster-type energy transfer [52,53] from Trp to the retinals in QuasAr1. The fluorescence quantum yields of Tyr and Trp in neutral water at 20 °C are ϕ_F_(Tyr) = 0.14 [55] and ϕ_F_(Trp) = 0.15 [56,57].

Normalized fluorescence excitation quantum distributions ${E^{\prime }}_{ex}(\lambda )$ of a fresh thawed QuasAr1 sample in pH 8 Tris buffer for fluorescence detection wavelengths λ_F,det_ in the range from 300 nm to 780 nm are shown in Figure S4 of the Supplementary Materials (section S4). They confirm the excitation wavelength dependent fluorescence emission of the dominant retinal component Ret_580 and other present retinal components.

### 2.2. Heating-Coling Cycle of a Fresh Thawed QuasAr1 Sample

**Figure 4:**
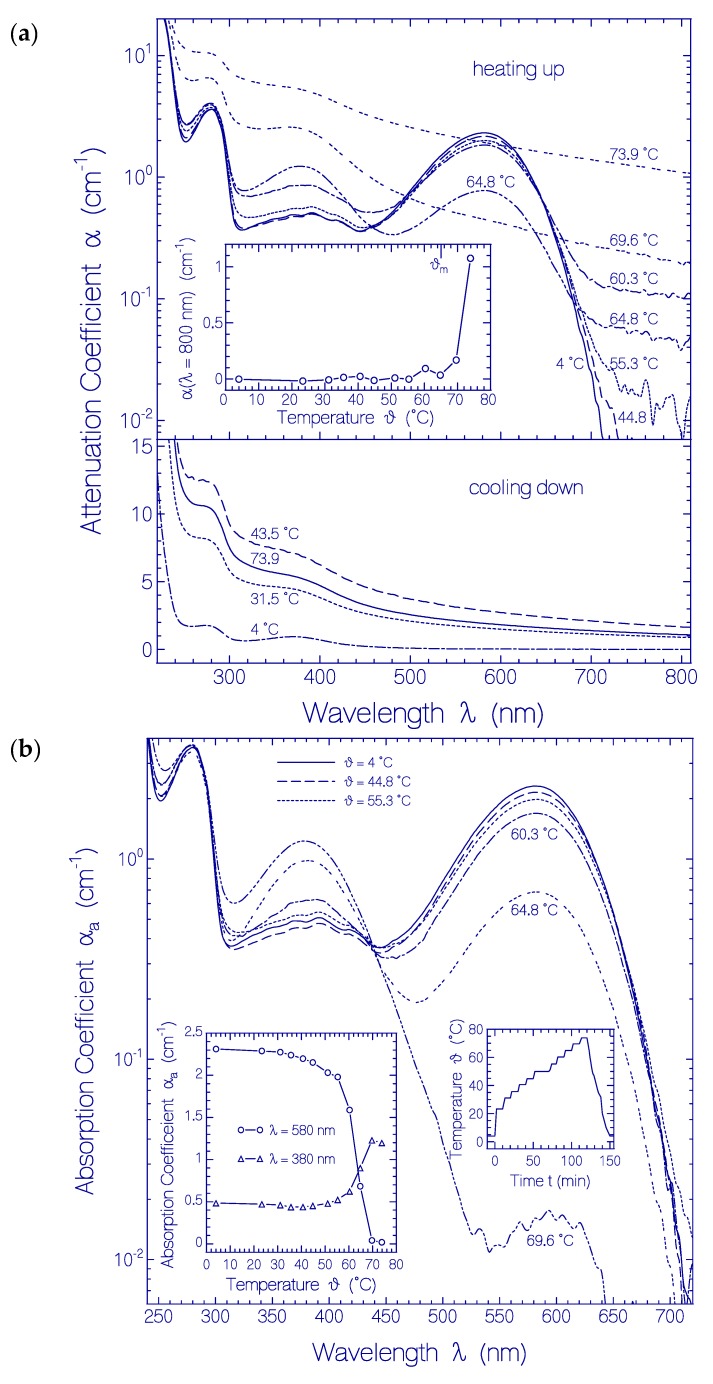
Heating-cooling cycle behavior of a fresh thawed QuasAr1 sample in pH 8 Tris buffer. (**a**) Attenuation coefficient spectra α(λ) development during stepwise sample heating up (top part) and cooling down (bottom part). Inset in top part: Temperature dependent attenuation coefficient development α(800 nm) during sample heating up. (**b**) Absorption coefficient spectra α_a_(λ) development during stepwise sample heating up. Left inset: Temperature dependent absorption coefficient development α_a_(580 nm) and α_a_(380 nm). Right inset: Applied heating and cooling temperature profile ϑ(*t*).

A fresh thawed sample of QuasAr1 was stepwise heated up to ϑ = 73.9 °C, and then cooled down. Thereby attenuation coefficient spectra were measured. The results are shown in Figure 4. The applied temporal heating and cooling temperature profile is depicted in the right inset of Figure 4b. The apparent QuasAr1 protein melting temperature ϑ_m_ was determined by the onset of a steep attenuation rise in the transparency spectral region of QuasAr1 [58] due to coalescing of denatured unfolded proteins [59]. The apparent protein melting temperature is an indicator of the protein thermal stability.

The temperature dependent development of attenuation coefficient spectra α(λ) of QuasAr1 is shown in Figure 4a. Up to about 55 °C the attenuation spectra remained nearly unchanged (see top part of Figure 4a). Then the attenuation band of Ret_580 decreased and a new attenuation band around 380 nm built up (Ret_380). Light scattering became detectable above 55 °C and increased strongly above 65 °C. The temperature dependence of the light attenuation in the transparency region of QuasAr1 at 800 nm during the sample heating up is shown in the inset of the top part of Figure 4a. The apparent protein melting temperature determined by the onset of steeply rising light attenuation (light scattering) is ϑ_m_ = 65 ± 3 °C. The light scattering increased during heating up to 73.9 °C, and continued to increase during cooling down to 43.5 °C (see bottom part of Figure 4a). Then the light attenuation decreased likely due to aggregated particle sedimentation (see attenuation curve belonging to 31.5 °C in bottom part of Figure 4a). The final attenuation curve (dash-dotted curve in bottom part of Figure 4a) was obtained after centrifugation of the sample for 20 min with 4400 rpm at 4 °C.

The main part of Figure 4b shows the temperature dependent development of absorption coefficient spectra α_a_(λ) of QuasAr1 during stepwise sample heating up (the attenuation coefficient spectra of Figure 4a were deprived of their scattering contribution, see procedure described in Section 4.2). The PRSB Ret_580 absorption band peaking around 580 nm decreased with rising temperature by deprotonation to RSB Ret_380 forming a new absorption band around 380 nm. For ϑ = 69.6 °C Ret_580 is nearly completely converted to Ret_380. Therefore the curve α_a_(λ, ϑ = 69.6 °C) in the wavelength range from ≈ 310 nm to ≈ 500 nm of Figure 4b represents the absorption coefficient spectrum of Ret_380. The absorption cross-section spectrum of Ret_380 is determined in section S3 of the Supplementary Materials from α_a_(λ, ϑ = 4 °C) and α_a_(λ, ϑ = 69.6 °C) of Figure 4b (dashed curve in Figure S3).

The left inset in Figure 4b displays the temperature dependent development of the absorption coefficients α_a_(ϑ) at λ = 580 nm (line-connected circles) and at λ = 380 nm (line-connected triangles). The curves clearly show the rising conversion of Ret_580 to Ret_380 with increasing temperature. The absorption at 380 nm below 40 °C is determined by the S_0_-S_n_ absorption of Ret_580 and the absorption of the already present deprotonated retinal Schiff base isomers of the fresh thawed unheated sample. In the stepwise sample heating the conversion of PRSB (Ret_580) to RSB (Ret_380) starts already at about ϑ = 40 °C and becomes very strong above ϑ = 55 °C well below the apparent protein melting temperature of ϑ_m_ ≈ 65 °C.

### 2.3. Temporal Development of QuasAr1 at Refrigerator Temperature of 2.5 °C

The thermal stability of QuasAr1 in pH 8 Tris buffer at ϑ = 2.5 ± 0.5 °C in the dark was studied by carrying out transmission spectra measurements over a duration of 80 days and by measuring fluorescence emission and fluorescence excitation spectra at the end of the storage time.

**Figure 5:**
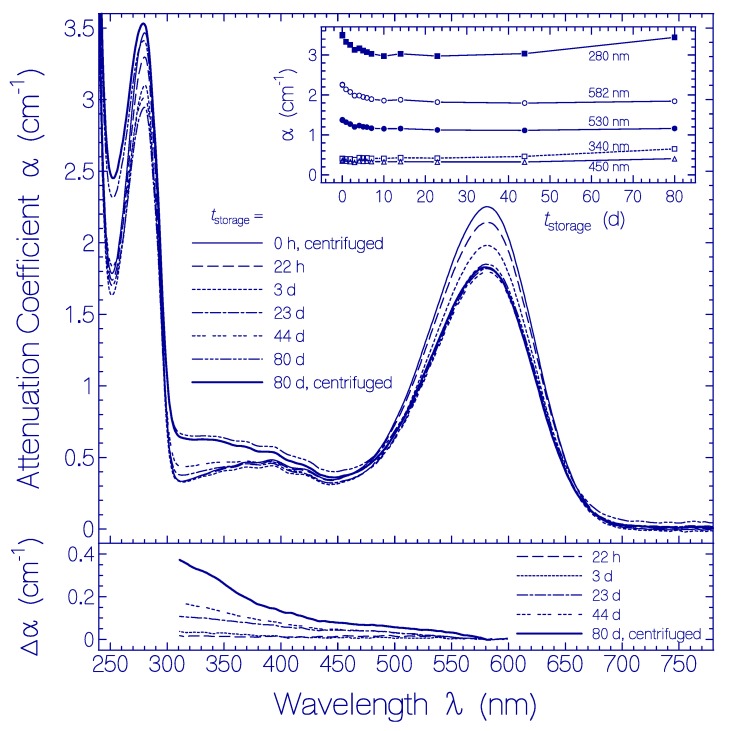
Temporal development of attenuation coefficient spectra α(λ,*t_storage_*) of QuasAr1 in pH 8 Tris buffer at 2.5 ± 0.5 °C in the dark. The storage times in the refrigerator are listed in the legend. The top inset shows attenuation coefficients at fixed wavelengths versus the storage time *t*_storage_. The bottom part displays difference attenuation spectra $\Delta \alpha (\lambda ,t_{storage})=\alpha (\lambda ,t_{storage})-\alpha (\lambda ,0)\left[\alpha (580\;nm,t_{storage})/\alpha (580\;nm,0)\right]$.

The temporal development of the attenuation coefficient spectra α(λ) is shown in Figure 5. In the top main part attenuation coefficient spectra are shown for selected storage times *t*_storage_. For *t*_storage_ = 80 days the attenuation spectra are shown before and after sample centrifugation to see the small light scattering contribution due to protein aggregation. The top inset shows the temporal attenuation coefficient development for some selected wavelengths. The bottom part displays difference attenuation spectra $\Delta \alpha (\lambda ,t_{storage})=\alpha (\lambda ,t_{storage})-\alpha (\lambda ,0)\left[\alpha (580\;nm,t_{storage})/\alpha (580\;nm,0)\right]$ for selected storage times.

Within the first ten days the attenuation coefficient of Ret_580 around 580 nm and the attenuation coefficient of the apoprotein around 280 nm decreased with time while the attenuation in the range from 310 nm to 470 nm remained approximately unchanged. The attenuation reduction around 580 nm and around 280 nm are attributed i) partly to the conversion of Ret_580 to other retinals absorbing in the 470–310 nm range (see differential attenuation coefficient spectra in the bottom part of Figure 5) and ii) partly to QuasAr1 tight small aggregate formation (specific surface reduction [60,61,62]) and/or loose aggregate cluster compactization with storage time (cluster size small therefore not showing up in light attenuation in the transparency spectral region; loosely packed globules with small volume fill factor densify to tightly packed globules, thereby the apparent absorption cross-section per molecule decreases because of specific surface reduction of the aggregates [63]). In the time range from *t*_storage_ = 10 d to 80 d the Ret_580 absorption band remained nearly unchanged. However, in this time range the apoprotein absorption changed. The main apoprotein absorption band around 280 nm increased and broadened (stronger absorption around 250 nm and around 310 nm). This behavior is attributed to dynamic QuasAr1 apoprotein restructuring. The increase of the apoprotein absorption strength is attributed to some increase of the oscillator strength of the S_0_ to S_1_ transition of Trp due to protein restructuring. In the wavelength range from 310 nm to 400 nm some absorption contribution from possibly formed dityrosine [64], tyrosinyl radicals [65], and tryptophanyl radicals [66] cannot be excluded [67]. The tryptophan involvement as chromophore element in photoreceptors is known for the UV-B photoreceptor UVR8 from *Arabidopsis thaliana* [68,69] (see also [51] with references therein) and for the LITE-1 photoreceptor in *Caenorhabditis elegans* [70,71].

Only a slight increase of light scattering was found after 80 days of storage by comparing the attenuation coefficient spectra measured before and after sample centrifugation (for 20 min at 4400 rpm). The slow spectral changes indicate the high thermal stability of QuasAr1 at 2.5 °C.

**Figure 6:**
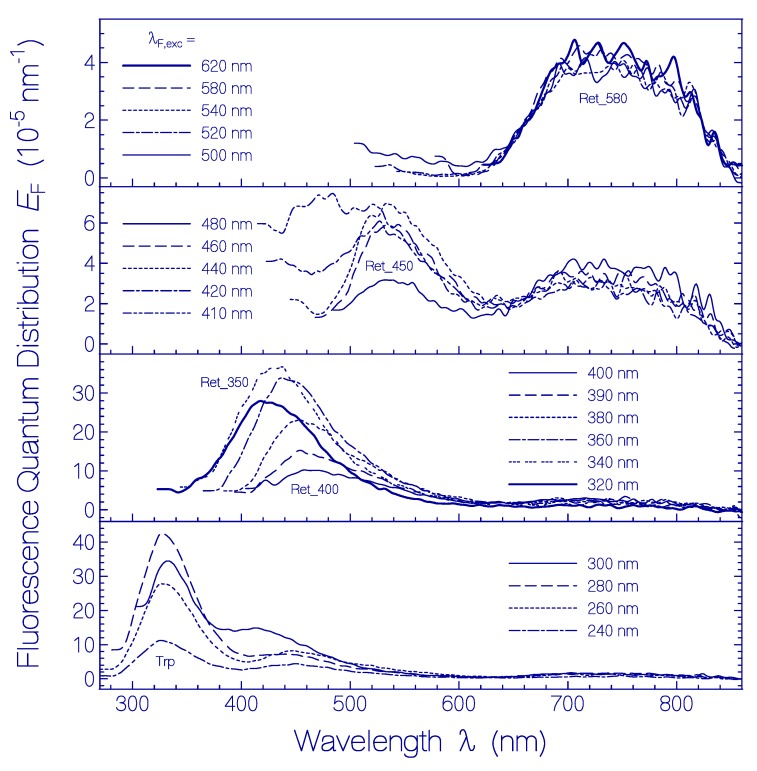
Fluorescence emission quantum distributions *E*_F_(λ) of QuasAr1 in pH 8 Tris buffer stored at 2.5 °C for a duration of 80 days. The fluorescence excitation wavelengths λ_F,exc_ are listed in the legends.

Fluorescence emission quantum distributions *E*_F_(λ) of QuasAr1 after 80 days of sample storage in the dark at 2.5 °C are shown in Figure 6. Fluorescence excitation in the wavelength range from λ_F,exc_ = 500 nm to 620 nm (top sub-figure) resulted in the fluorescence emission band of Ret_580 with fluorescence maximum around 740 nm. Sample excitation in the wavelength range from λ_F,exc_ = 420 nm to 480 nm (second top sub-figure) revealed a second fluorescence emission band with emission maximum around 540 nm. It is attributed to a PRSB isomer named Ret_450. In the second lowest sub-figure fluorescence emission spectra in the excitation wavelength region from λ_F,exc_ = 320 nm to 400 nm are displayed. A weak fluorescence band peaking around 470 nm is resolved for fluorescence excitation around λ_F,exc_ ≈ 400 nm (RSB, Ret_400). A stronger fluorescence band peaking around 440 nm is observed for fluorescence excitation around λ_F,exc_ ≈ 350 nm (RSB, Ret_350). A short-wavelength fluorescence band with maximum around 330 nm, in the bottom sub-figure of Figure 6, belongs to the apoprotein Trp emission (absorption band maximum around λ_F,exc_ ≈ 280 nm). Fluorescence excitation in the wavelength range from λ_F,exc_ = 240 nm to 280 nm additionally caused fluorescence emission around 450 nm. This indicates excitation energy transfer from apoprotein absorbing species Tyr and Trp to Ret_350.

The excitation wavelength dependence of the total fluorescence quantum yield ϕ_F_ of QuasAr1 in pH 8 Tris buffer after 80 days of storage at 2.5 °C in the dark is displayed by the line-connected circle curve in Figure 3. The spectral changes of ϕ_F_(λ_F,exc_) due to sample storage are seen easily by comparison with the ϕ_F_(λ_F,exc_) curve a fresh thawed QuasAr1 sample (dashed-line connected triangle curve in Figure 3). For λ_F,exc_ < 500 nm the fluorescence quantum yield is increased by the thermally formed retinal isomers named Ret_450, Ret_400, and Ret_350 and by the thermally induced apoprotein restructuring with fluorescence emission of Trp (λ_F,exc_ around 280 nm).

Normalized fluorescence excitation spectra of QuasAr1 in pH 8 Tris buffer stored in the dark at 2.5 °C for 80 days are presented in section S5 of the Supplementary Materials (Figure S5). They confirm the thermal formation of retinal isomers with increased fluorescence efficiency compared to Ret_580 and the Trp fluorescence emission.

### 2.4. Temporal Development of QuasAr1 at Room Temperature

The thermal stability of QuasAr1 in pH 8 Tris buffer at room temperature (ϑ = 21–25 °C) in the dark was studied by carrying out transmission spectra measurements over a duration of 101 days and by measuring fluorescence emission and fluorescence excitation spectra after 50 days and at the end of the storage time.

**Figure 7:**
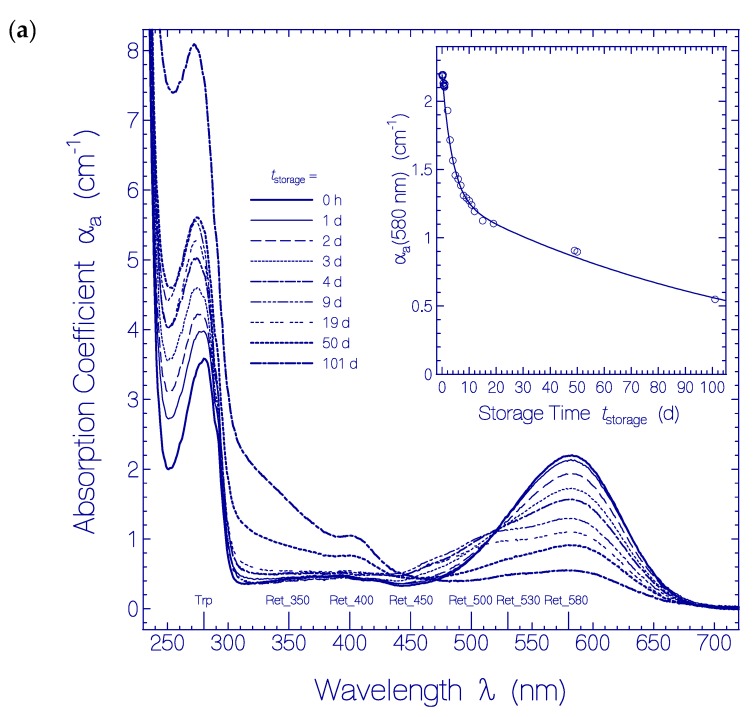

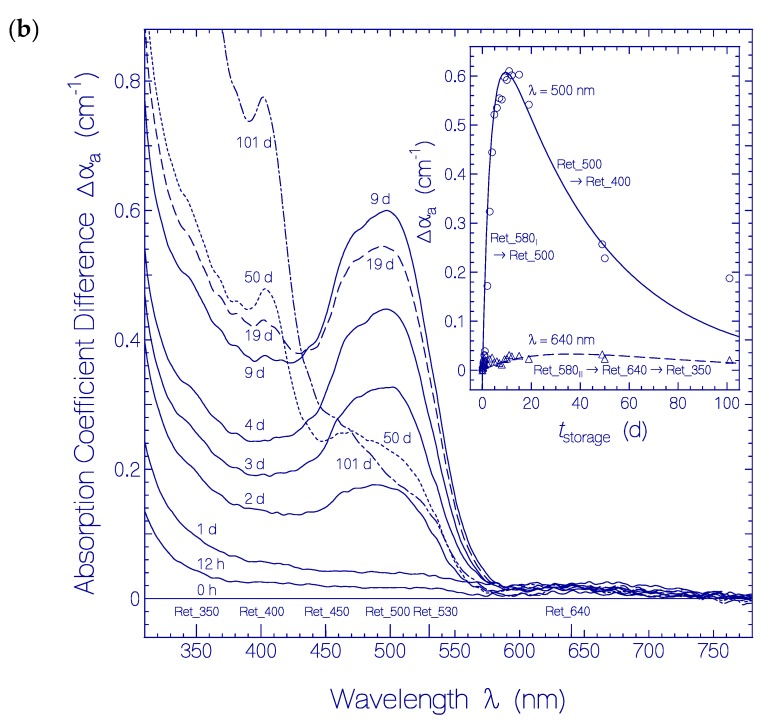
(**a**) Temporal development of absorption coefficient spectra α_a_(λ) of QuasAr1 in pH 8 Tris buffer stored in the dark at room temperature (ϑ = 21–25 °C). The storage times are listed in the legend (*t*_storage_ = 0 h refers to absorption coefficient spectrum measurement immediately after sample thawing). The inset shows the temporal development of α_a_(580 nm) where the circles are data points and the solid curve is a two-component single exponential fit according to $\alpha_{a,580\;nm}(t_{storage})=\alpha_{a,580\;nm}(0)\left[\kappa_{{\mathrm{Ret}\_580}_{I}}\exp(-t_{storage}/\tau_{\mathrm{Ret}\_580,I})+\kappa_{{\mathrm{Ret}\_580}_{\mathrm{II}}}\exp(-t_{storage}/\tau_{\mathrm{Ret}\_580,\mathrm{II}})\right]$ with α_a,580 nm_(0) = 2.193 cm^−1^, $\kappa_{{\mathrm{Ret}\_580}_{I}}$ = 0.41, $\tau_{{\mathrm{Ret}\_580}_{I}}$ = 3.8 d, $\kappa_{{\mathrm{Ret}\_580}_{\mathrm{II}}}$ = 0.59, and $\tau_{{\mathrm{Ret}\_580}_{\mathrm{II}}}$ = 120 d. (**b**) Temporal development of the corresponding difference absorption coefficient spectra $\Delta \alpha_{a}(\lambda ,t_{storage})=\alpha_{a}(\lambda ,t_{storage})-\alpha_{a,\mathrm{Ret}\_580}(\lambda ,t_{storage})-\alpha_{a,\mathrm{residual}\;\mathrm{retinals}}(\lambda ,0)$. The inset shows Δα_a_ at λ = 500 nm and λ = 640 nm versus storage time *t*_storage_. The Δα_a_ data at λ = 500 nm are fitted by $\Delta \alpha_{a,500\;nm}(t_{storage})=\Delta \alpha_{a,500\;nm,\max}\left[1-\exp\left(-t_{storage}/\tau_{{\mathrm{Ret}\_580}_{I}}\right)\right]\exp\left(-t_{storage}/\tau_{PT,I}\right)$ with $\Delta \alpha_{a,500\;nm,\max}$ = 0.83 cm^−1^, $\tau_{{\mathrm{Ret}\_580}_{{}_{1}}}$= 3.8 d, and τ_PT,I_ = 42 d. The Δα_a_ data at λ = 640 nm are fitted by $\Delta \alpha_{a,640\;nm}(t_{storage})=\Delta \alpha_{a,640\;nm,\max}\left[1-\exp\left(-t_{storage}/\tau_{{\mathrm{Ret}\_580}_{\mathrm{II}}}\right)\right]\exp\left(-t_{storage}/\tau_{PT,II}\right)$ with $\Delta \alpha_{a,640\;nm,\max}$ = 0.3 cm^−1^, $\tau_{{\mathrm{Ret}\_580}_{{}_{1I}}}$ = 120 d, and τ_PT,II_ = 42 d.

The measured attenuation coefficient spectra α(λ) are presented in Figure S6 of the Supplementary Materials (Section S6). In Figure 7a the temporal development of the absorption coefficient spectra α_a_(λ) is shown. The curves were derived from Figure S6 by removing the scattering contributions α_s_(λ) according to $\alpha_{a}(\lambda )=\alpha (\lambda )-\alpha_{s}(\lambda )$ with $\alpha_{s}(\lambda )=\alpha_{s}(\lambda_{0})\times {\left(\lambda_{0}/\lambda \right)}^{\gamma }$ whereby λ_0_ was set to 800 nm and γ was adjusted in the transparency region (see below Section 4.2). The storage times are listed in the legend. The wavelength positions of maximum absorption of the originally present species (Ret_580 and Trp) and the formed species (Ret_530, Ret_500, Ret_450, Ret_400, Ret_350) are indicated.

In the main part of Figure 7a it is seen that the absorption decreased around 580 nm (PRSB, Ret_580), and new absorption built-up and decreased around 500 nm (PRSB, Ret_500). The absorption increased with storage time around 400 nm (RSB, Ret_400) and around 350 nm (RSB, Ret_350). The temporal increase of absorption below 320 nm is attributed to apoprotein restructuring with Trp enlarged absorption oscillator strength.

The inset of Figure 7a shows the dependence of the absorption coefficient α_a_(580 nm) versus storage time *t*_storage_ (circles are experimental data). The decrease of the absorption coefficient with storage time is fitted by a two-component single exponential decay function according to:

(1) $$\alpha_{a,580\;nm}(t_{storage})=\alpha_{a,580\;nm}(0)\left[\kappa_{{\mathrm{Ret}\_580}_{I}}\exp(-t_{storage}/\tau_{\mathrm{Ret}\_580,I})+\kappa_{{\mathrm{Ret}\_580}_{\mathrm{II}}}\exp(-t_{storage}/\tau_{\mathrm{Ret}\_580,\mathrm{II}})\right]$$

In Equation (1) α_a,580 nm_(0) is the total initial absorption coefficient at *t*_storage_ = 0. $\kappa_{{\mathrm{Ret}\_580}_{I}}$ is the fraction of Ret_580 with fast absorption decay time constant τ_Ret_580,I_. This component is named Ret_580_I_. $\kappa_{{\mathrm{Ret}\_580}_{\mathrm{II}}}=1-\kappa_{{\mathrm{Ret}\_580}_{I}}$ is the fraction of Ret_580 with slow absorption decay time constant τ_Ret_580,II_. This component is named Ret_580_II_. The fit parameters are α_a,580 nm_(0) = 2.193 cm^−1^, $\kappa_{{\mathrm{Ret}\_580}_{I}}$ = 0.41, $\tau_{{\mathrm{Ret}\_580}_{I}}$ = 3.8 d, $\kappa_{{\mathrm{Ret}\_580}_{\mathrm{II}}}$ = 0.59, and $\tau_{{\mathrm{Ret}\_580}_{\mathrm{II}}}$ = 120 d.

In Figure 7b the temporal development of absorption coefficient spectra of new formed species (Ret_640, Ret_530, Ret_500, Ret_450, Ret_400, Ret_350) are displayed. In the main part of Figure 7b the Ret_580 contribution α_a,Ret_580_(λ,*t*_storage_) and the original residual retinal contributions α_a, residual retinals_(λ) are subtracted from α_a_(λ,*t*_storage_) of Figure 7a, i.e.:

(2) $$\Delta \alpha_{a}(\lambda ,t_{storage})=\alpha_{a}(\lambda ,t_{storage})-\alpha_{a,\mathrm{Ret}\_580}(\lambda ,t_{storage})-\alpha_{a,\mathrm{residual}\;\mathrm{retinals}}(\lambda ,0)$$

is displayed. The curves show i) formation of a weak absorption band around 640 nm (formation of PRSB isomer Ret_640), ii) build-up and decrease of a broad absorption band around 500 nm (formation of PRSB Ret_500), iii) build-up of an absorption band around 400 nm (formation of RSB isomer Ret_400), iv) build-up of an absorption band around 350 nm (formation of RSB isomer Ret_350), and v) build-up of long-wavelength apoprotein absorption in the range < 340 nm. The Ret_500 absorption band changed its shape for long-time sample storage *t*_storage_ ≥ 50 d. Shoulders are seen around λ ≈ 530 nm (Ret_530) and around λ ≈ 450 nm (Ret_450). They may be due to new formed retinal isomer forms or due to Ret_500 isomer position shift due to apoprotein adjacent structure changes. The minor part of the PRSB isomer Ret_580 (fraction $\kappa_{{\mathrm{Ret}\_580}_{I}}$, named Ret_580_I_, likely a *cis* isomer) is converted dominantly to the short wavelength absorbing PRSB Ret_500 (likely a *trans* isomer), and the dominant part of the PRSB isomer Ret_580 (fraction $\kappa_{{\mathrm{Ret}\_580}_{\mathrm{II}}}$, named Ret_580_II_, likely a *trans* isomer) is converted to the long-wavelength absorbing PRSB isomer Ret_640 (likely a *cis* isomer). Ret_500 is thought to deprotonate to Ret_400 (likely a RSB *trans* isomer), and Ret_640 is thought to deprotonate to Ret_350 (likely a RSB *cis* isomer).

The inset in Figure 7b displays the temporal development of Δα_a_(500 nm) (circles, initial build-up of Ret_500 due to conversion of Ret_580_I_ to Ret_500 and subsequent decrease of Ret_500 due to conversion to Ret_400) and the temporal development of Δα_a_(640 nm) (triangles, weak build-up of Ret_640 due to conversion of Ret_580_II_ to Ret_640 and concurrent conversion of Ret_640 to Ret_350). The temporal development of Δα_a_(500 nm) is fitted by:

(3) $$\Delta \alpha_{a,500\;nm}(t_{storage})=\Delta \alpha_{a,500\;nm,\max}\left[1-\exp\left(-t_{storage}/\tau_{{\mathrm{Ret}\_580}_{I}}\right)\right]\exp\left(-t_{storage}/\tau_{PT,I}\right)$$

Δα_a,500 nm,max_ is the expected maximum Δα_a_(500 nm) for *t*_storage_ → ∞ in the absence of deprotonation (τ_PT,I_ → ∞). $\tau_{{\mathrm{Ret}\_580}_{I}}$ is the decay time constant of Ret_580_I_. τ_PT,I_ is the time constant of Ret_500 deprotonation. The fit parameters are Δα_a,500 nm,max_ = 0.83 cm^−1^, $\tau_{{\mathrm{Ret}\_580}_{I}}$ = 3.8 d, and τ_PT,I_ = 42 d. (Δα_a,500 nm_ at *t*_storage_ = 101 d is larger than the fit value since short-wavelength absorption bands extend to λ = 500 nm and Δα_a,500nm_(101 d) is not only due to Ret_500 absorption).

The temporal development of Δα_a_(640 nm) is fitted by:

(4) $$\Delta \alpha_{a,640\;nm}(t_{storage})=\Delta \alpha_{a,640\;nm,\max}\left[1-\exp\left(-t_{storage}/\tau_{{\mathrm{Ret}\_580}_{\mathrm{II}}}\right)\right]\exp\left(-t_{storage}/\tau_{PT,II}\right)$$

Δα_a,640 nm,max_ is the expected maximum Δα_a_(640 nm) for *t*_storage_ → ∞ in the absence of deprotonation (τ_PT,II_ → ∞). τ_Ret_580,II_ is the decay time constant of Ret_580_II_. τ_PT,II_ is the time constant of Ret_640 deprotonation. The fit parameters are Δα_a,640 nm,max_ = 0.3 cm^−1^, $\tau_{{\mathrm{Ret}\_580}_{\mathrm{II}}}$ = 120 d, and τ_PT,II_ = 42 d.

**Figure 8:**
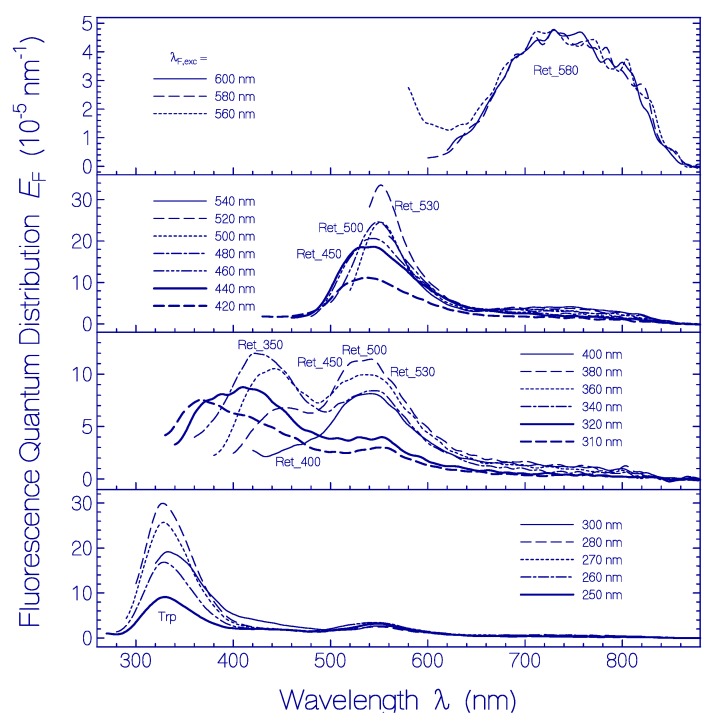
Fluorescence emission quantum distributions *E*_F_(λ) of QuasAr1 in pH 8 Tris buffer stored at room temperature (ϑ = 21–25 °C) for a duration of 50 days. The fluorescence excitation wavelengths λ_F,exc_ are listed in the legends.

Fluorescence emission quantum distributions *E*_F_(λ) of QuasAr1 after 50 days of sample storage in the dark at room temperature (ϑ = 21–25 °C) are shown in Figure 8. Fluorescence excitation in the wavelength range of λ_F,exc_ ≥ 560 nm (top sub-figure) resulted in the fluorescence emission band of Ret_580 with fluorescence maximum around 740 nm. Sample excitation in the region from λ_F,exc_ = 540 nm to λ_F,exc_ = 420 nm (second top sub-figure) resulted in peak fluorescence emission in the range from 550 nm to 530 nm. This emission is dominantly attributed to the formed protonated retinal Schiff base isomers Ret_530, Ret_500, and Ret_450. In the second lowest sub-figure fluorescence emission spectra are resolved resulting from the deprotonated retinal Schiff base isomers Ret_400 (λ_F,exc_ ≈ 400 nm, λ_F,max_ ≈ 470 nm, weak fluorescence emission) and Ret_350 (λ_F,exc_ ≈ 350 nm, λ_F,max_ ≈ 430 nm). In the bottom sub-figure the fluorescence emission peaking around λ_F,max_ ≈ 330 nm is due to the apoprotein Trp emission (either directly excited or populated by excitation transfer from photo-excited Tyr to Trp).

In Figure S7 of the Supplementary Materials (Section S7) the fluorescence emission quantum distributions of QuasAr1 after 101 days of storage in the dark at room temperature are shown. The reduced presence of weakly fluorescing Ret_580 increased the overall fluorescence quantum yield in the wavelength region from 420 nm to 540 nm (stronger fluorescent Ret_530 and Ret_450).

**Figure 9:**
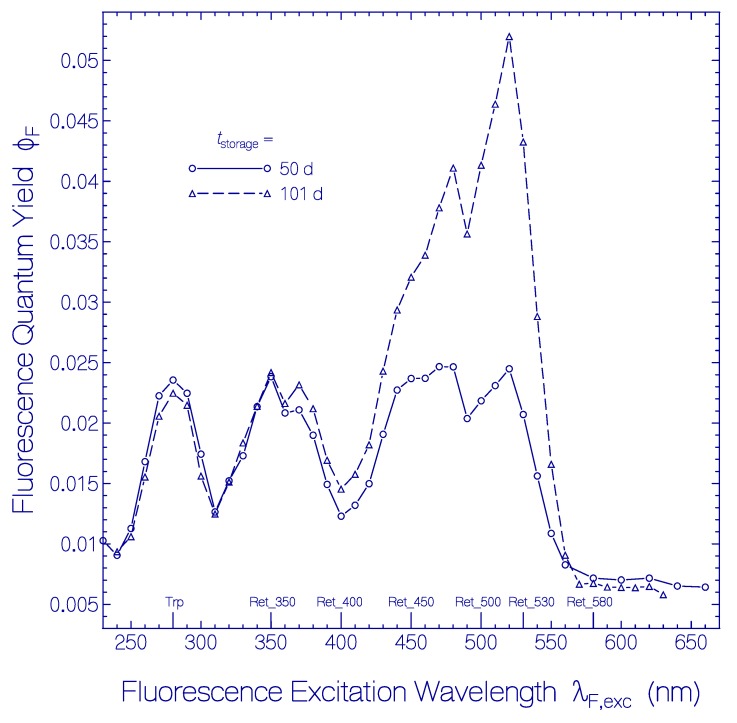
Dependence of the total fluorescence quantum yield ϕ_F_ on fluorescence excitation wavelength λ_F,exc_ for QuasAr1 in pH 8 Tris buffer stored in the dark at room temperature (ϑ = 21–25 °C) for durations of 50 days and 101 days.

The excitation wavelength dependence of the fluorescence quantum yield ϕ_F_ of QuasAr1 in pH 8 Tris buffer after 50 days and after 101 days of storage in the dark at room temperature is displayed in Figure 9. ϕ_F_ is the ratio of the total amount of emitted fluorescence photons to the total amount of absorbed photons from the various absorbing species *i* in QuasAr1 at the selected excitation wavelength λ_F,exc_. This means:

(5) $$\phi_{F}(\lambda_{F,exc})=\sum_{i}\frac{\alpha_{a,i}(\lambda_{F,exc})}{\alpha_{a}(\lambda_{F,exc})}\phi_{F,i}\left(\lambda_{F,exc}\right)$$

where *i* runs over the species absorbing at λ_F,exc_ with the absorption coefficients α_a,i_(λ_F,exc_), ϕ_F,i_(λ_F,exc_) is the fluorescence quantum yield of component *i*, and $\alpha_{a}(\lambda_{F,exc})=\sum_{i}\alpha_{a,i}(\lambda_{F,exc})$ is the total absorption coefficient at λ_F,exc_. The wavelength positions of the absorption band maxima of the present components, protonated retinal Schiff bases Ret_580, Ret_530, Ret_500, Ret_450, deprotonated retinal Schiff bases Ret_400 and Ret_350, and the apoprotein contribution Trp are indicated at the bottom of Figure 9. The fluorescence quantum yield contributions of Ret_500 and Ret_400 are not well resolved. The fluorescence quantum yield of Ret_580 of ϕ_F_ ≈ 0.007 is the same after 50 days and 101 days of storage as of the fresh sample immediately after thawing. Ret_530, Ret_450, and Ret_350 are a factor of 5 to 10 stronger fluorescent than Ret_580. The fluorescence quantum yield of the apoprotein ϕ_F,Trp_ is reduced by apoprotein excitation energy transfer to the retinal isomers.

Normalized fluorescence excitation spectra of QuasAr1 in pH 8 Tris buffer stored in the dark at room temperature for 50 days (Figure S8) and 101 days (Figure S9) are presented in section S8 of the Supplementary Materials. They confirm the thermal formation of retinal isomers with increased fluorescence efficiency compared to Ret_580 and the thermal apoprotein restructuring with increased absorption strength. The formation of small amounts of strongly fluorescent protonated retinal Schiff base isomers Ret_530 and Ret_450 by thermal activated ground-state isomerization of Ret_580 are resolved in Figures S8 and S9.

## 3. Discussion

In Section 2 we reported spectroscopic investigation of QuasAr1 absorption and emission at pH 8 in Tris buffer. The samples were studied under different conditions: i) fresh thawed samples, ii) thermally aged samples at refrigerated temperature (2.5 °C) and room temperature (21–25 °C), and iii) heat-denaturized samples. The measurements provided information on the thermal protein stability, the presence of different original apoprotein structures, the original retinal isomer composition, the thermal induced isomer conformation changes, the protonated retinal Schiff base isomers (PRSB) proton release to deprotonated retinal Schiff base isomers (RSB), and the thermal apoprotein restructuring of the originally present apoprotein structures showing up in UV spectral changes and absorption strength increase. The formation of new protonated retinal Schiff base isomers and their deprotonation to retinal Schiff base isomers occurred in parallel with the dynamic opsin apoprotein restructuring.

In the heterologous expression of QuasAr1 the retinal cofactor is covalently bound to the opsin protein via a lysine Schiff base. It is dominantly present in protonated form. About 86% of retinal was found to be present as protonated retinal Schiff base (PRSB) Ret_580, and about 14% were found to be present mainly as neutral retinal Schiff base (RSB) isomers Ret_400 and Ret_350 and small amounts of other protonated retinal Schiff base isomers as Ret_450 (see Figure 1 and section S4 of the Supplementary Materials).

At refrigerator temperature (≈ 2.5 °C) over a period of 80 days only small conversion of Ret_580 to Ret_500, Ret_450, Ret_400 and Ret_350 was observed. Some apoprotein restructuring showed up in increased UV absorption strength.

At room temperature (≈ 23 °C) within the observation time of 101 days formation of new PRSB isomers (Ret_640, Ret_530, Ret_500, Ret_450) and significant deprotonation of the PRSB isomers to RSB isomers (Ret_640 to Ret_350, Ret_500 to Ret_400) occurred together with apoprotein restructuring showing up in increased UV absorption. The temporal two-component single exponential absorption decrease of Ret_580 (Figure 7a) indicated its composition of two main isomer components Ret_580_I_ (likely a PRSB *cis* isomer in a specific QuasAr1 amino acid residue arrangement Apoprotein_initial,I_) and Ret_580_II_ (likely a PRSB *trans* isomer in another specific QuasAr1 amino acid residue arrangement Apoprotein_initial,II_). The temporal formation and decay of the absorption coefficient spectra of new formed species (Figure 7b) indicated i) a ground-state thermal activated isomerization of Ret_580_I_ to Ret_500 (likely a PRSB *trans* isomer) and the subsequent deprotonation of Ret_500 to Ret_400 (likely a RSB *trans* isomer), and ii) a ground-state thermal activated isomerization of Ret_580_II_ to Ret_640 (likely a PRSB *cis* isomer) and the concurrent deprotonation of Ret_640 to Ret_350 (likely a RSB *cis* isomer). QuasAr1 sample heating above 60 °C resulted in fast PRSB chromophore deprotonation to RSB. The thermal studies indicated energy barrier involved ground-state isomerizations, irreversible protein restructuring with irreversible protonated retinal Schiff base deprotonation and intrinsic apoprotein residue (mainly Trp) rearrangement with increased absorption oscillator strength.

**Figure 10:**
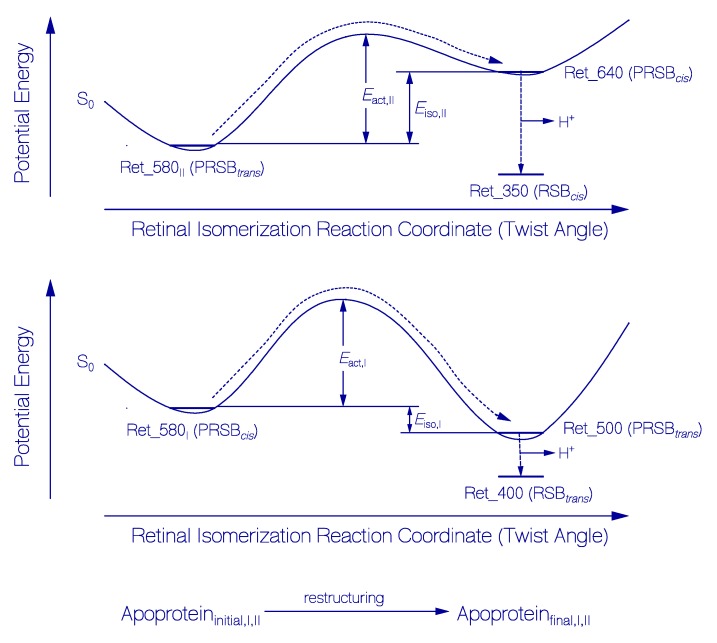
Schematic reaction coordinate diagrams for thermal activated S_0_ ground-state protonated retinal Schiff base isomerizations and apoprotein restructuring assisted irreversible deprotonations to retinal Schiff base isomers. Top part: Isomerization of Ret_580_II_ to Ret_640 and subsequent deprotonation to Ret_350. Middle part: Isomerization of Ret_580_I_ to Ret_500 and subsequent deprotonation to Ret_400. Bottom part: Parallel occurring opsin restructuring of originally present apoprotein structures (Apoprotein_initial,I_ and Apoprotein_initial,II_) to final apoprotein structures (Apoprotein_final,I_ and Apoprotein_final,II_) of QuasAr1 acting on protonated retinal Schiff base isomerization and deprotonation.

The protonated retinal Schiff base ground-state isomerization, protonated retinal Schiff base deprotonation, and the apoprotein restructuring dynamics are illustrated in Figure 10. The top part illustrates the isomerization of the PRSB Ret_580_II_ isomer (likely all-*trans* isomer in a specific QuasAr1 protein conformation Apoprotein_II_) to the PRSB Ret_640 isomer (likely 13-*cis* isomer in the same specific QuasAr1 protein conformation) and the concurrent proton release from Ret_640 to the stable formation of Ret_350 (likely a RSB 13-*cis* isomer). The middle part illustrates the isomerization of the PRSB Ret_580_I_ isomer (likely 13-*cis* isomer in a specific QuasAr1 protein conformation Apoprotein_I_ ) to the PRSB Ret_500 isomer (likely all-*trans* isomer in the same specific QuasAr1 protein conformation) and the subsequent proton release from Ret_500 to the stable formation of Ret_400 (likely a RSB all-*trans* isomer). The bottom part illustrates the concurrent occurring apoprotein restructuring supporting the ground-state protonated retinal Schiff base isomerizations and deprotonations.

The energetic level positions *E*_iso,I_ of Ret_500 and *E*_iso,II_ of Ret_640 may be estimated from the expected maximum absorption differences Δα_a,500 nm, max_ and Δα_a,640 nm, max_ of Equations (3) and (4). Assuming equal absorption cross-sections σ_a,Ret_580_(580 nm), σ_a,Ret_500_(500 nm), and σ_a,Ret_640_(640 nm), the fractions χ_Ret_500_ and χ_Ret_640_ of thermally populated Ret_500 and Ret_640 would be [54]:

(6) $$\chi_{\mathrm{Ret}\_500}=\frac{\Delta \alpha_{a,500\;nm,\max}}{\alpha_{a,580\;nm}(0)\kappa_{\mathrm{Ret}\_580,I}}$$

(7) $$\chi_{\mathrm{Ret}\_640}=\frac{\Delta \alpha_{a,640nm,\max}}{\alpha_{a,580\;nm}(0)\kappa_{\mathrm{Ret}\_580,\mathrm{II}}}$$

The energy level positions *E*_iso,I_ and *E*_iso,II_ are obtained by application of the Boltzmann level position law [72]:

(8) $$\chi_{\mathrm{Ret}\_500}=\frac{\exp\left(-E_{iso,I}/(k_{B}\vartheta )\right)}{1+\exp\left(-E_{iso,I}/(k_{B}\vartheta )\right)}$$

and:

(9) $$\chi_{\mathrm{Ret}\_640}=\frac{\exp\left(-E_{iso,II}/(k_{B}\vartheta )\right)}{1+\exp\left(-E_{iso,II}/(k_{B}\vartheta )\right)}$$

where *k*_B_ is the Boltzmann constant and ϑ is the temperature.

Solving Equations (8) and (9) for *E*_iso,I_ and *E*_iso,II_ gives:

(10) $$E_{iso,I}=-\ln\left(\frac{\chi_{\mathrm{Ret}\_500}}{1-\chi_{\mathrm{Ret}\_500}}\right)k_{B}\vartheta$$

and:

(11) $$E_{iso,II}=-\ln\left(\frac{\chi_{\mathrm{Ret}\_640}}{1-\chi_{\mathrm{Ret}\_640}}\right)k_{B}\vartheta$$

Insertion of parameters gives: χ_Ret_500_ = 0.92 (Δα_a,500 nm,max_ = 0.83 cm^−1^, α_a,580 nm_(0) = 2.193 cm^−1^, κ_Ret_580,I_ = 0.41), χ_Ret_640_ = 0.23 (Δα_a,500 nm,max_ = 0.3 cm^−1^, α_a,580 nm_(0) = 2.193 cm^−1^, κ_Ret_580,II_ = 0.59), *E*_iso,I_ = −9.98 × 10^−21^ J = −500 cm^−1^ × *hc*_0_ (*k*_B_ = 1.38 × 10^−23^ J K^−1^, ϑ = 296 K is temperature, *h* is the Planck constant, and *c*_0_ is the speed of light in vacuum), and *E*_iso,II_ = 4.94 × 10^−21^ J = 248 cm^−1^ × *hc*_0_.

The time constants of Ret_580_I_ isomerization to Ret_500, τ_Ret_580,I_, of Ret_580_II_ isomerization to Ret_640, τ_Ret_580,II_, of Ret_500 deprotonation to Ret_400, τ_PT,I_, and of Ret_640 deprotonation to Ret_350, τ_PT,II_, were determined above (Equations (3) and (4)). They are related to the energy activation barriers *E*_act,I_, *E*_act,II_, *E*_act,PT,I_, and *E*_act,PT,II_ by the Arrhenius relation [73] according to:

(12) $$\tau_{\mathrm{Ret}\_580,I}=\tau_{0}\exp\left(\frac{E_{act,I}}{k_{B}\vartheta }\right)$$

(13) $$\tau_{\mathrm{Ret}\_580,\mathrm{II}}=\tau_{0}\exp\left(\frac{E_{act,II}}{k_{B}\vartheta }\right)$$

(14) $$\tau_{PT,i}=\tau_{0}\exp\left(\frac{E_{act,PT,i}}{k_{B}\vartheta }\right),\;i\;=\;I,\;II$$

where $\tau_{0}=h/(k_{B}\vartheta )$ is the attempt time constant of barrier crossing [59,74]. Solving Equations (12)–(14) for the activation energy barriers gives:

(15) $$E_{act,I}=\ln\left(\frac{\tau_{\mathrm{Ret}\_580,I}}{\tau_{0}}\right)k_{B}\vartheta$$

(16) $$E_{act,II}=\ln\left(\frac{\tau_{\mathrm{Ret}\_580,\mathrm{II}}}{\tau_{0}}\right)k_{B}\vartheta$$

(17) $$E_{act,PT,i}=\ln\left(\frac{\tau_{PT,i}}{\tau_{0}}\right)k_{B}\vartheta .\;i=I,\;II$$

Insertion of parameters (ϑ = 296 K, τ_0_ ≈ 1.6 × 10^−13^ s, τ_Ret_580,I_ = 3.8 d, τ_Ret_580,I_ = 120 d, τ_PT,I_ ≈ τ_PT,II_ ≈ 42 d) leads to *E*_act,I_ = 1.72 × 10^−19^ J = 8670 cm^−1^ × *hc*_0_, *E*_act,II_ = 1.86 × 10^−19^ J = 9380 cm^−1^ × *hc*_0_, and *E*_act,PT,I_ ≈ *E*_act,PT,II_ ≈ 1.82 × 10^−19^ J ≈ 9160 cm^−1^ × *hc*_0_.

The performed data analysis of fresh thawed and of heat treated QuasAr1 allowed to determine the absorption cross-section spectra of PRSB Ret_580 (a composition of PRSB 13-*cis* isomer Ret_580_I_ and PRSB all-*trans* isomer Ret_580_II_ in two different protein adjacent amino acid arrangements Apoprotein_initial,I_ and Apoprotein_initial,II_) and of the RSB Ret_380 (likely unresolved composition of Ret_350 and Ret_400) which are shown in Figure S3. Knowledge of the absorption cross-section spectra allowed the determination of the radiative lifetimes τ_rad_ of the S_1_–S_0_ emission transitions using the Strickler–Berg formula according to [75,76,77]:

(18) $$\tau_{rad}=\frac{n_{A}{\bar{\lambda }}_{F}^{3}}{8\pi c_{0}n_{F}^{3}\bar{\sigma }}$$

where *n*_A_ and *n*_F_ are the average refractive indices of the aqueous buffer solution in the S_0_–S_1_ absorption band region and the S_1_–S_0_ emission band region, respectively, and *c*_0_ is the velocity of light in vacuum. ${\bar{\lambda }}_{F}={\left(\int_{em}E_{F}(\lambda )\lambda^{3}d\lambda /\int_{em}E_{F}(\lambda )d\lambda \right)}^{1/3}$ is the average S_1_–S_0_ fluorescence emission wavelength, and ${\bar{\sigma }}_{a}=\int_{abs}\left(\sigma_{a}(\lambda )/\lambda \right)d\lambda$ is the absorption cross-section strength of the S_0_–S_1_ absorption band. Using appropriate absorption cross-section data from Figure S3, fluorescence quantum distribution data from Figure 6 and refractive indices of water, we determine τ_rad_(Ret_580) = 9.32 ns (*n*_F_ = 1.33, *n*_A_ = 1.3328, ${\bar{\lambda }}_{F}$ = 745 nm, ${\bar{\sigma }}_{a}$ = 3.33 × 10^−17^ cm^2^) and τ_rad_(Ret_380) ≈ 3.74 ns (*n*_F_ = 1.3366, *n*_A_ = 1.3406, ${\bar{\lambda }}_{F}$ ≈ 460 nm, ${\bar{\sigma }}_{a}$ = 1.94 × 10^−17^ cm^2^).

Average Strickler-Berg based fluorescence lifetimes τ_F,SB_ are obtained from the radiative lifetimes and the fluorescence quantum yields according to:

(19) $$\tau_{F,SB}=\phi_{F}\tau_{rad}$$

The obtained values are τ_F,SB_(Ret_580) ≈ 61.5 ps (ϕ_F_ ≈ 0.0065) and τ_F,SB_(Ret_380) ≈ 150 ps (ϕ_F_ ≈ 0.04).

The fluorescence quantum yields and the fluorescence lifetimes of the protonated retinal Schiff base chromophore Ret_580 of fresh thawed QuasAr1 and of the thermally formed deprotonated retinal Schiff base Ret_380 are extraordinary large compared to the parent wild-type Archaerhodopsin 3 (ϕ_F_(Arch) = 9 × 10^−4^) [36,42]. The performed mutations on Arch to get QuasAr1 led to a slowing down of the excited-state isomerization dynamics via twisted internal conversion (S_1_–S_0_ conical intersection). They cause some restriction (barrier) along the reactive coordinate (twist angle) of photoisomerization. The slower relaxation along the S_1_ state potential energy surface towards the funnel position of S_1_ to S_0_ internal conversion leads to the broad-band fluorescence emission of increased efficiency and longer fluorescence lifetime ([51] and references therein). In Table S1 of the Supplementary Materials (section S9) absorption, fluorescence, and primary photoisomerization parameters of some rhodopsins are collected for comparison.

## 4. Materials and Methods 

### 4.1. Sample Preparation

QuasAr1 gene was a gift from Adam E. Cohen (Addgene plasmid # 64135, [42]). *E. coli* optimized gene was cloned into pET21a(+) vector between the NdeI and SalI restriction sites with a C-terminal TEV protease cleavage site and a HIS_6_ tag (ENLYFQSLVDLEHHHHHH).

The expression plasmid (pet21a+) carrying QuasAr1 was transformed into C41(DE3) *E. coli* cells. To induce the protein expression we used 0.5 mM isopropyl β-D-thiogalactopyranoside (IPTG; Carl Roth GmbH, Karlsruhe, Germany) and the LB media was supplemented with 5 µM all-trans retinal (ATR; Sigma-Aldrich, St. Louis, USA). The cells were incubated at 37 °C for 4 h and then harvested. The cells were disrupted using an EmulsiFlex-C3 Homogenizer (AVESTIN Inc., Ottawa, Canada). The membrane fraction was collected by ultracentrifugation (45,000 rpm) for 1 h at 4 °C (Type 45 Ti; Beckman Inc., Indianapolis, USA) and then resuspended in buffer containing 50 mM Tris-HCl (pH 8.0), 300 mM NaCl, 0.1 mM phenylmethanesulfonyl fluoride (PMSF), 1.5% n-dodecyl-β-D-maltoside (DDM, GLYCON Biochemicals GmbH, Luckenwalde, Germany), and 0.3% cholesteryl hemisuccinate (CHS, Sigma-Aldrich, St. Louis, USA) and stirred overnight for solubilization. The insoluble fraction was removed by ultracentrifugation (200,000 × g, 1 h at 4 °C). The QuasAr1 protein was purified by Ni-NTA affinity and using an ÄKTAxpress protein purification system (GE Healthcare Life Science, Chicago, USA) configured with a HisTrap HP Ni-NTA column. The protein was collected in the final buffer containing 50 mM Tris-HCl (pH 8.0), 150 mM NaCl, 0.02% DDM, 0.004% CHS, 0.1 mM PMSF, and 5% glycerol. 

The expressed QuasAr1 protein in the final buffer was aliquoted to amounts of 30 μL in Eppendorf tubes, shock-frozen, and stored at –80 °C until thawing for experimental investigations.

### 4.2. Spectroscopic Measurements

Transmission measurements, *T*(λ) (λ is wavelength), were carried out with a spectrophotometer (Cary 50, Varian Australia Pty Ltd, Mulgrave, Victoria, Australia). Attenuation coefficient spectra were calculated by the relation, α(λ)=−ln[T(λ)]/l, were *l* is the sample length. In the case of negligible protein light scattering the attenuation coefficient spectrum α(λ) is equal to the absorption coefficient spectrum α_a_(λ). Otherwise it comprises absorption (α_a_) and scattering (α_s_) contributions according to $\alpha (\lambda )=\alpha_{a}(\lambda )+\alpha_{s}(\lambda )$. The scattering coefficient spectrum is approximated by the empirical relation [78] $\alpha_{s}(\lambda )=\alpha_{s}(\lambda_{0}){\left(\lambda_{0}/\lambda \right)}^{\gamma }$ where the wavelength λ_0_ is selected in the transparency region and γ ≤ 4 is fitted to the experimental attenuation in the transparency region. Absorption coefficient spectra became available by subtracting the scattering contribution from the measured attenuation coefficient spectra.

The QuasAr1 melting was studied by stepwise sample heating up, then cooling down and thereby measuring the attenuation coefficient spectra development [58,59]. The apparent protein melting temperature ϑ_m_ was derived from the onset of strong light attenuation in the transparency region of QuasAr1.

The thermal stability of QuasAr1 at room temperature (21–25 °C) and refrigerator temperature (2.5 ± 0.5 °C) was determined by storing QuasAr1 samples at these temperatures in the dark and measuring transmission spectra at certain time intervals.

Fluorescence spectroscopic measurements were carried out with a spectrofluorimeter (Cary Eclipse, Varian Australia Pty Ltd, Mulgrave, Victoria, Australia). Fluorescence emission quantum distributions *E*_F_(λ) were determined from fluorescence emission spectra measurements at fixed excitation wavelengths [52,79,80]. The dye rhodamine 6G in methanol was used as reference standard for fluorescence quantum distribution calibration (fluorescence quantum yield ϕ_F,ref_ = 0.93 [81]). The fluorescence quantum yield ϕ_F_ was calculated using the relation $\phi_{F}=\int_{em}E_{F}(\lambda )d\lambda$ where the integration runs over the fluorescence emission wavelength region. Fluorescence excitation quantum distributions *E*_ex_(λ) were recorded by scanning the fluorescence excitation wavelength over the absorption wavelength region at fixed fluorescence detection wavelengths [82]. Magic angle conditions were applied for the fluorescence recordings (vertical polarized excitation and orientation of the fluorescence detection polarizer at an angle of 54.7° to the vertical [83]). The spectra were corrected for the spectral sensitivity of the spectrometer and the photodetector.

## 5. Conclusions

The rhodopsin fluorescent voltage sensor QuasAr1 [42] was characterized by its absorption and emission spectroscopic behavior and its long-time thermal stability. At refrigerator temperature it may be used over a period of about 40 days without significant absorption and fluorescence spectroscopic changes. At room temperature it is possible to store the voltage sensor over about one day without severe retinal Schiff base deprotonation and opsin protein restructuring.

In the dark at room temperature the formation of new retinal isomers in the ground-state of QuasAr1 took place by thermal overcoming of energy barriers and by lowering the potential energy levels of the protonated Schiff base isomers (dominantly Ret_500 and Ret_640) and the deprotonated Schiff base isomers (dominantly Ret_400 and Ret_350) due to dynamic protein restructuring on a time scale of days. The isomerization dynamics of QuasAr1 retinals in the excited state (photoisomerization) occurs on a ten picosecond timescale due to a different barrier-involved S_1_ state potential energy surface structure.

## Supplementary Materials

Supplementary Materials can be found at https://www.mdpi.com/1422-0067/20/17/4086/s1. References [84,85,86,87,88,89,90,91,92,93,94,95,96,97,98,99,100,101,102,103] are cited in the supplementary materials

## Abbreviations

Arch	Archaerhodopsin 3 from *Halorubrum sodomense*
GECI	Genetically encoded calcium indicator
GEVI	Genetically encoded voltage indicator
PRSB	Protonated retinal Schiff base
QuasAr	Quality superior to Arch
Ret_xxx	Retinal with absorption maximum approximately at xxx nm
RSB	Retinal Schiff base
Trp	Tryptophan
Tyr	Tyrosine
VSD	Voltage sensing domain
