# Supplementary material for: Absorption and Emission Spectroscopic Investigation of the Thermal Dynamics of the Archaerhodopsin 3 Based Fluorescent Voltage Sensor QuasAr1
Source: Int J Mol Sci. 2019 Aug 21;20(17):4086. doi: 10.3390/ijms20174086 (PMC6747118; doi:10.3390/ijms20174086)
Supplement: Supplementary file 1 [file ijms-20-04086-s001.pdf]

International Journal of *Molecular Sciences*

MDPI

Supplementary Materials: Absorption and Emission Spectroscopic Investigation of the Thermal Dynamics of the Archaerhodopsin 3 Based Fluorescent Voltage Sensor QuasAr1

Alfons Penzkofer [1,*], Arita Silapetere [2] and Peter Hegemann [2]

S1. Amino Acid Sequence of QuasAr1

The amino acid sequence of the here investigated mutated Arch protein QuasAr1 is displayed in Figure S1. The apoprotein molar mass is M_{apo} = 25.548 kDa. It contains 11 phenylalanine (Phe, F), 7 tryptophan (Trp, W), and 12 tyrosine (Tyr, Y) residues.

```
        10         20         30         40         50         60
MDPIALQAGY DLLGDGRPET LWLGIGTLLM LIGTFYFLVR GWGVTDKDAR EYYAVTILVS

        70         80         90        100        110        120
GIASAAYLSM FFGIGLTEVS VGGEMLDIYY ARYAHWLFTT PLLLLHLALL AKVDRVTIGT

       130        140        150        160        170        180
LVGVDALMIV TGLIGALSHT AIARYSWWLF STICMIVVLY VLATSLRSAA KERGPEVAST

       190        200        210        220        230        240
FNTLTALVLV LWTAYPILWI IGTEGAGVVG LGIETLLFMV LDVTAKVGFG FILLRSRAIL

       250        260        270
GDTEAPEPSA GADENLYFQS LVDLEHHHHH H
```

Figure S1. Amino acid sequence of QuasAr1 (Arch P60S, T80S, D95H, D106H, F161V).

S2. Structural Formulae of Retinal Schiff Bases

In Figure S2 structural formulae of protonated retinal Schiff base (PRSB) isomers and unprotonated retinal Schiff base (RSB) isomers are shown.

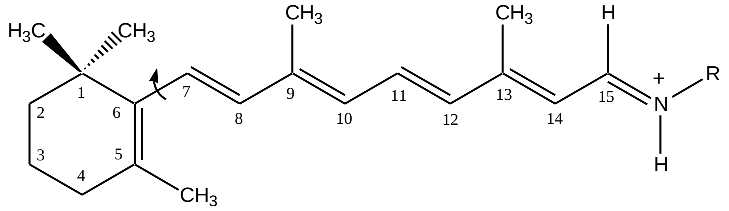

Protonated retinal Schiff base all-trans,15-anti

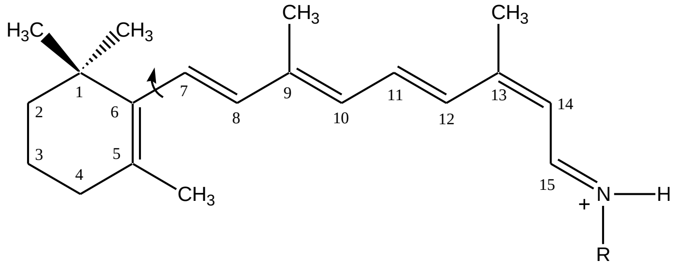

Protonated retinal Schiff base 13-cis,15-anti

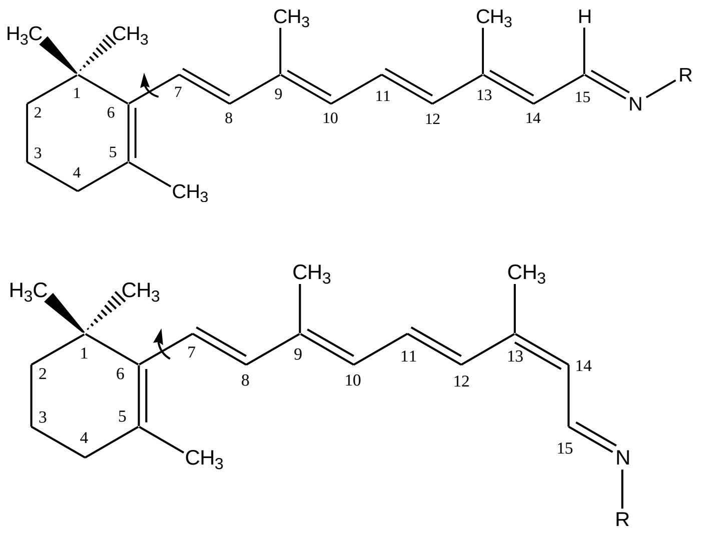

Retinal Schiff base all-trans,15-anti

Retinal Schiff base 13-cis,15-anti

Figure S2. Structural formulae of some protonated retinal Schiff and neutral retinal Schiff base isomers.

S3. Determination of Absorption Cross-section Spectra of Ret_580 and Ret_380

The molecule absorption cross-section spectra are derived from the molecule absorption coefficient spectra by the relation $\sigma_a(\lambda) = \alpha_a(\lambda)/N$ where N is the molecule number density. The absorption coefficient α_a(280 nm) of QuasAr1 of Figure 1 is composed of apoprotein and retinal contributions according to α_a(280 nm) = $\alpha_{apoprotein}$(280 nm) + $\alpha_{retinal}$(280 nm) = 3.73 cm^{-1}. The retinal contribution is set to be $\alpha_{retinal}$(280 nm) = 0.37 cm^{-1} [51] giving $\alpha_{apoprotein}$(280 nm) = 3.36 cm^{-1}. The apoprotein absorption coefficient is given by $\alpha_{apoprotein} = N_{apoprotein} \times \sigma_{apoprotein}$(280 nm) where $N_{apoprotein}$ is the apoprotein number density. The apoprotein absorption cross-section is composed of the contribution of 11 Phe (σ_{Phe}(280 nm) = 3.8×10^{-21} cm^2 [84]), 7 Trp (σ_{Trp}(280 nm) = 2.10×10^{-17} cm^2 [84]), and 12 Tyr (σ_{Tyr}(280 nm) = 4.62×10^{-18} cm^2 [84]) giving $\sigma_{apoprotein}$(280 nm) = 2.025×10^{-16} cm^2 and $N_{apoprotein}$ = 1.66×10^{16} cm^{-3}. The retinal number density $N_{retinal}$ is equal to the apoprotein number density (one retinal isomer is covalently bound to one opsin apoprotein) and is composed of the number density N_{Ret_580} of protonated retinal Schiff base Ret_580 and the number density $N_{residual\ retinals}$ of residual retinal components according to $N_{retinal} = N_{Ret\_580} + N_{residual\ retinals} = N_{apoprotein}$. The ratio of the number densities $N_{residual\ retinals}/N_{Ret\_580}$ is approximated by the ratio of S_0-S_1 absorption coefficient integrals $\int_{310\,nm}^{465\,nm} \alpha_{a,\text{residual retinals}}(\tilde{\nu})d\tilde{\nu} \Big/ \int_{440\,nm}^{700\,nm} \alpha_{a,\text{Ret\_580}}(\tilde{\nu})d\tilde{\nu}$ ($\tilde{\nu} = \lambda^{-1}$ is wavenumber) of Figure 1 giving a value of $N_{residual\ retinals} = 0.16 \times N_{Ret\_580} = (0.16/1.16) \times N_{retinal} = 2.3 \times 10^{15}$ cm^{-3} and $N_{Ret\_580} = N_{retinal}/(1 + 0.16) = 1.43 \times 10^{16}$ cm^{-3}. The resulting absorption cross-section spectrum $\sigma_{a,Ret\_580}(\lambda) = \alpha_{a,Ret\_580}(\lambda)/N_{Ret\_580}$ is displayed by the solid curve in Figure S3.

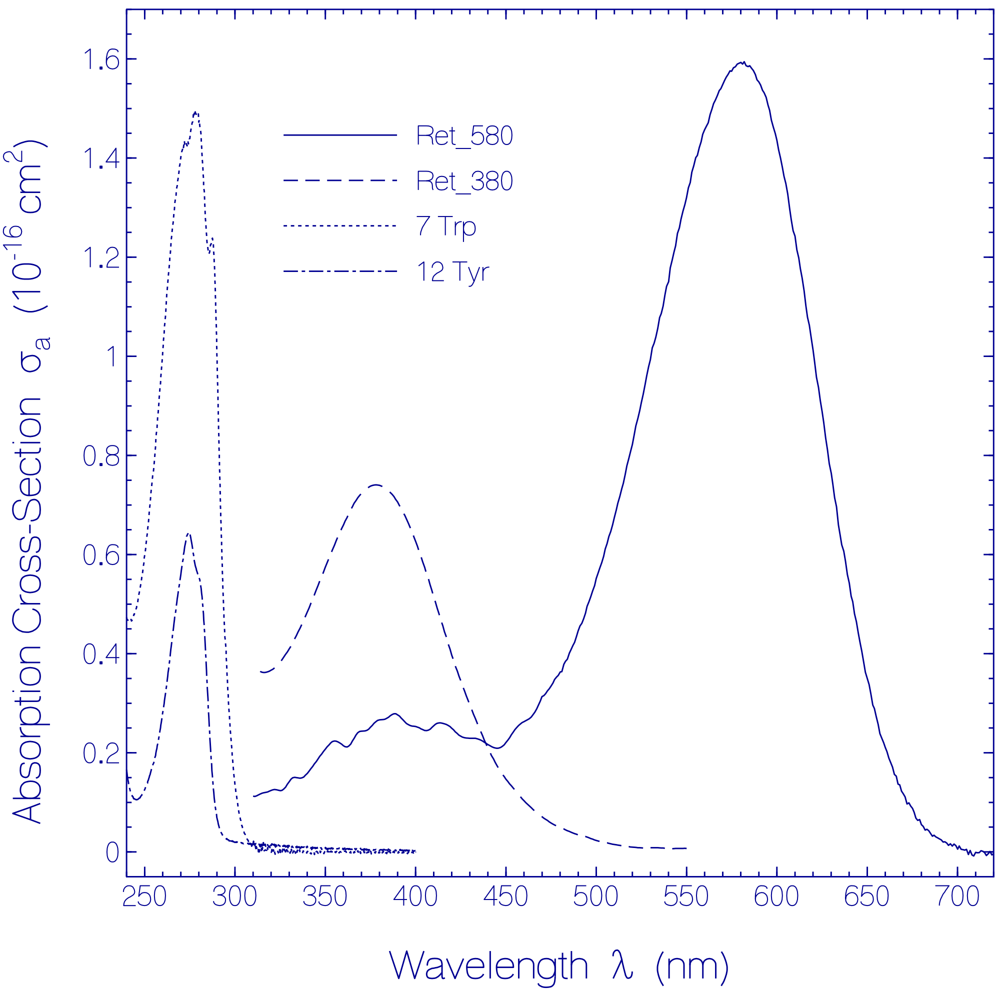

Figure S3. Absorption cross-section spectra. Solid curve: Ret_580 (PRSB) of QuasAr1 in pH 8 Tris buffer (determined from Figure 1 and estimated Ret_580 number density) Dashed curve: Ret_380 (RSB, determined form heat degraded sample at 69.6 °C of Figure 4b). Dotted curve: absorption cross-section spectrum of 7 Trp molecules (taken from [84]). Dash-dotted curve: absorption cross-section spectrum of 12 Tyr molecules (taken from [84]).

The S_0–S_1 absorption cross-section integral of Ret_580 is $\sigma_{\mathrm{a,Ret\_580,int}} = \int_{440\,nm}^{700\,nm} \sigma_{\mathrm{a,Ret\_580}}(\tilde{\nu})d\tilde{\nu}$ = 6.1×10^{-13} cm. Its oscillator strength is given by [85]

$$f_{\mathrm{Ret\_580}} = 1.1296\times10^{12}\,\frac{9n}{(n^2+2)^2}\,\sigma_{\mathrm{a,Ret\_580,int}} \tag{S1}$$

where n is the refractive index averaged over the absorption band (here $n \approx n_{\mathrm{water}} \approx 1.333$). Insertion of parameters gives $f_{\mathrm{Ret\_580}} = 0.58$. The full spectral half-width of the S_0–S_1 absorption band of Ret_580 is $\delta\tilde{\nu}_{\mathrm{Ret\_580}} = 3600\ \mathrm{cm}^{-1}$.

The absorption cross-section spectrum of the deprotonated retinal Schiff base Ret_380 is shown by the dashed curve in Figure S3. It was obtained from the absorption coefficient spectrum $\alpha_a(\lambda, \vartheta = 69.6\ °\mathrm{C})$ of Figure 4b and the assumption that practically all PRSB Ret_580 is deprotonated to Ret_380 so that the number density of Ret_380 is now $N_{\mathrm{Ret\_380}} \approx N_{\mathrm{retinal}} \approx 1.66\times10^{16}\ \mathrm{cm}^{-3}$. The S_0-S_1 absorption cross-section integral of Ret_380 is $\sigma_{\mathrm{a,Ret\_380,int}} \approx \int_{310\,nm}^{550\,nm} \sigma_{\mathrm{a,Ret\_380}}(\tilde{\nu})d\tilde{\nu}$ = 5.2×10^{-13} cm. Its oscillator strength is

$$f_{\mathrm{Ret\_380}} = 1.1296\times10^{12}\,\frac{9n}{(n^2+2)^2}\,\sigma_{\mathrm{a,Ret\_380,int}} \tag{S2}$$

giving $f_{\mathrm{Ret\_380}}$ ≈ 0.49 ($n \approx n_{\mathrm{water}} \approx 1.34$). The full spectral half-width of the S_0-S_1 absorption band of Ret_380 is $\delta\tilde{\nu}_{\mathrm{Ret\_380}}$ = 7200 cm^{-1}.

The S_0–S_1 absorption cross-section integral of tyrosine is $\sigma_{\mathrm{Tyr,int}} \approx \int_{243\,nm}^{300\,nm} \sigma_{Tyr}(\tilde{\nu})d\tilde{\nu}$ = 1.87×10^{-14} cm, and the oscillator strength of tyrosine is $f_{Tyr} = 1.1296\times10^{12}\left(9n/(n^2+2)^2\right)\sigma_{\mathrm{Tyr,int}}$ = 0.0175 (n_{water}(280 nm) = 1.353).

The S_0–S_1 absorption cross-section integral of tryptophan is $\sigma_{\mathrm{Trp,int}} \approx \int_{237\,nm}^{320\,nm} \sigma_{Trp}(\tilde{\nu})d\tilde{\nu}$ = 1.162 × 10^{-13} cm, and the oscillator strength of tryptophan is $f_{Tyr} = 1.1296\times10^{12}\left(9n/(n^2+2)^2\right)\sigma_{\mathrm{Tyr,int}}$ = 0.1089.

S4. Normalized Fluorescence Excitation Quantum Distributions of Fresh Thawed QuasAr1

In Figure S4 normalized fluorescence excitation quantum distributions $E'_{ex}(\lambda) = E'_{ex,\lambda_{F,\mathrm{det}}}(\lambda)$ of a fresh thawed QuasAr1 sample in pH 8 Tris buffer are shown for fluorescence detection wavelengths $\lambda_{F,\mathrm{det}}$ in the range from 300 nm to 780 nm. The normalization is made so that $E'_{ex,\lambda_{F,\mathrm{det}}=720\,nm}(\lambda = 582\ nm)$ is equal to $\alpha_a(\lambda$ = 582 nm), i.e. $E'_{ex,\lambda_{F,\mathrm{det}}}(\lambda) = E_{ex,\lambda_{F,\mathrm{det}}}(\lambda)\alpha_a(582\ nm)/E_{ex,\lambda_{F,\mathrm{det}}=720\,nm}(\lambda = 582\ nm)$. $E_{ex,\lambda_{F,\mathrm{det}}}(\lambda)$ is given by $E_{ex,\lambda_{F,\mathrm{det}}}(\lambda) = \gamma_\alpha S_{\lambda_{F,\mathrm{det}}}(\lambda)/S_{exc}(\lambda)$ where $S_{\lambda_{F,\mathrm{det}}}(\lambda)$ is the detected fluorescence signal at $\lambda_{F,\mathrm{det}}$ as a function of the excitation wavelength λ, $S_{\mathrm{exc}}(\lambda)$ is the excitation light spectrum, and $\gamma_\alpha = [\alpha_a(\lambda)l + \alpha_a(\lambda_{F,\mathrm{det}})d]/\{1-\exp[-\alpha_a(\lambda)l - \alpha_a(\lambda_{F,\mathrm{det}})d]\}$ is an absorption correction factor (*l* is the sample length along the excitation direction, *d* is the sample width along the fluorescence detection direction) [80,82].

The curve $E'_{ex,\lambda_{F,\mathrm{det}}=720\,nm}(\lambda)$ represents the absorption coefficient spectrum of Ret_580. It was applied to determine $\alpha_{a,\mathrm{Ret\_580}}(\lambda)$ in Figure 1 and it was used in Figure S3 to determine the absorption cross-section spectrum of Ret_580. The normalized fluorescence excitation spectra for $\lambda_{F,\mathrm{det}}$ = 600 nm to 410 nm indicate the presence of retinal isomers absorbing in the wavelength region from 500 nm to 310 nm. The normalized fluorescence excitation spectra for $\lambda_{F,\mathrm{det}}$ = 370 nm to 300 nm indicate the QuasAr1 apoprotein absorption caused by Trp and Tyr.

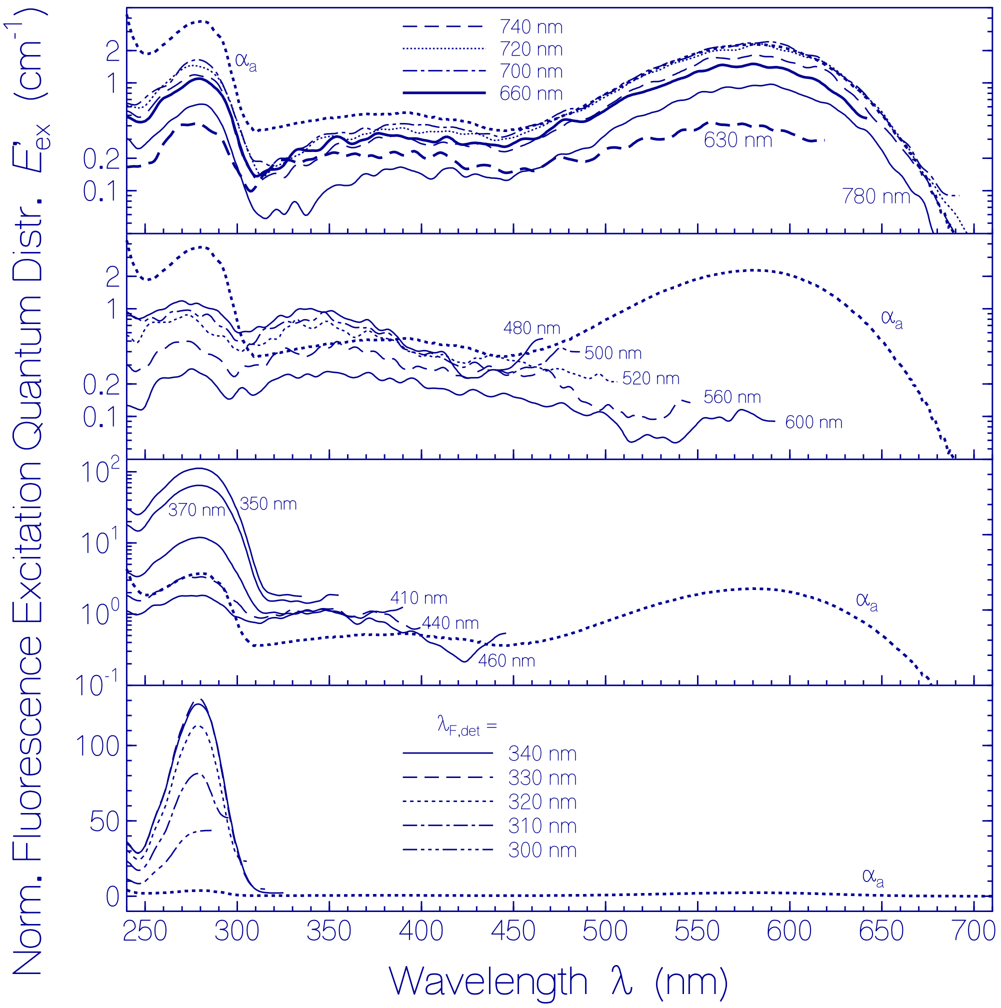

Figure S4. Normalized fluorescence excitation quantum distributions $E'_{ex,\lambda_{F,det}}(\lambda) = E_{ex,\lambda_{F,det}}(\lambda) \times \alpha_a(582\ nm) / E_{ex,\lambda_{F,det}=720\ nm}(582\ nm)$ of fresh thawed QuasAr1 in pH 8 Tris buffer for the fluorescence detection wavelengths $\lambda_{F,det}$ listed in the sub-figures. The thick dotted curves show the absorption coefficient spectrum $\alpha_a(\lambda)$ of the investigated sample.

S5. Normalized Fluorescence Excitation Quantum Distributions of QuasAr1 Stored at 2.5 °C

Normalized fluorescence excitation quantum distributions $E'_{ex}(\lambda) = E'_{ex,\lambda_{F,det}}(\lambda) = E_{ex,\lambda_{F,det}}(\lambda)\alpha_a(582\ nm) / E_{ex,\lambda_{F,det}=720\ nm}(\lambda = 582\ nm)$ of QuasAr1 in pH 8 Tris buffer stored in the dark at 2.5 °C for 80 days are shown in Figure S5 for different fluorescence detection wavelengths $\lambda_{F,det}$. For comparison the absorption coefficient spectrum $\alpha_a(\lambda)$ of the QuasAr1 sample stored in the dark for 80 days is included in the sub-figures.

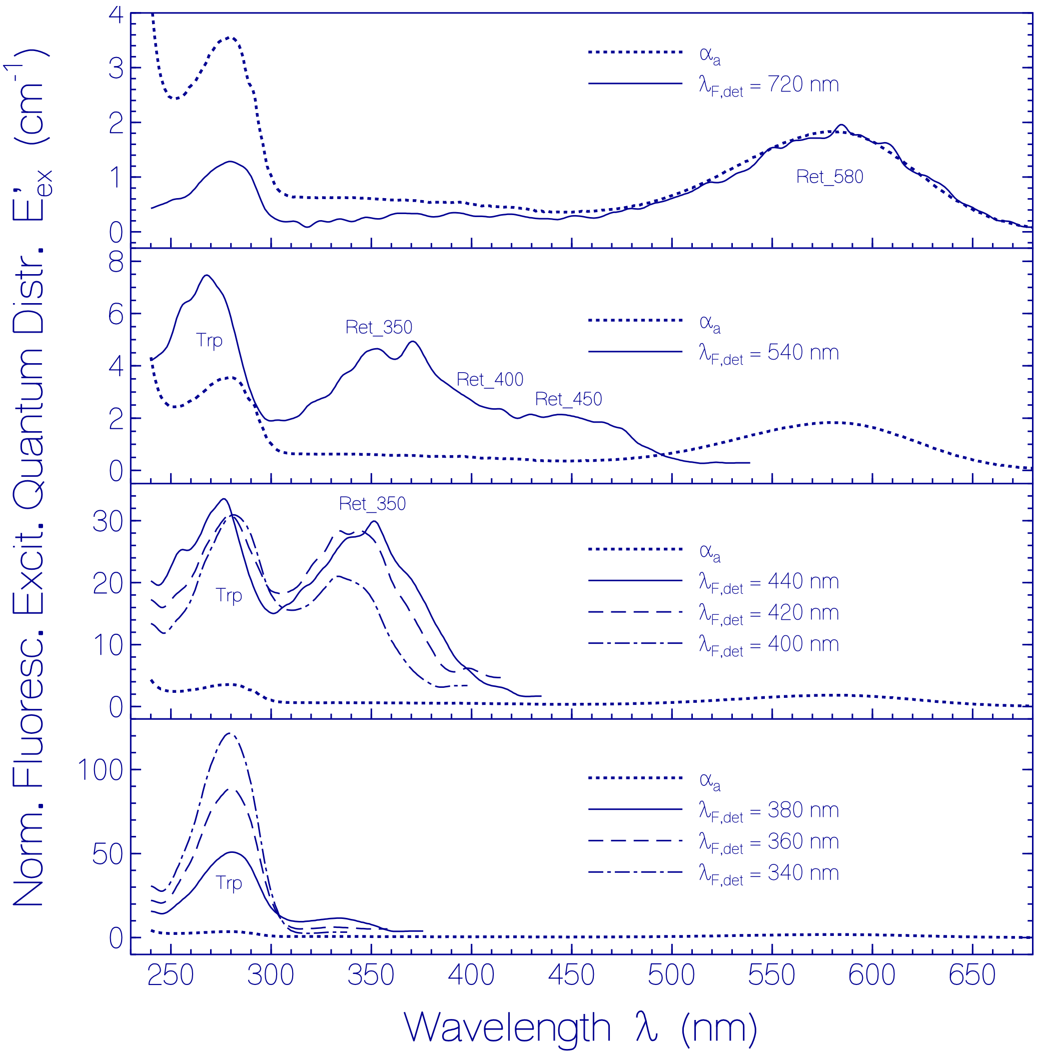

Figure S5. Normalized fluorescence excitation quantum distributions $E'_{ex,\lambda_{F,\det}}(\lambda) = E_{ex,\lambda_{F,\det}}(\lambda) \times \alpha_a(582\ nm) / E_{ex,\lambda_{F,\det}=720\ nm}(582\ nm)$ of QuasAr1 in pH 8 Tris buffer stored in the dark at 2.5±0.5 °C over a period of 80 days. The fluorescence detection wavelengths $\lambda_{F,det}$ are listed in the legends.

In the top sub-figure $E'_{ex,\lambda_{F,\det}=720\ nm}(\lambda)$ resembles the absorption coefficient spectrum of Ret_580 in the wavelength range λ ≥ 310 nm. For λ < 310 nm $E'_{ex,\lambda_{F,\det}=720\ nm}(\lambda)$ is dominated by apoprotein Tyr and Trp absorption and subsequent Förster-type energy transfer to Ret_580.

In the second-top sub-figure $E'_{ex,\lambda_{F,\det}=540\ nm}(\lambda)$ is displayed. It is determined by fluorescence emission at 540 nm from Ret_450, Ret_400, and Ret_350. $E'_{ex,\lambda_{F,\det}=540\ nm}(\lambda < 310\ nm)$ is dominated by photo-excitation of apoprotein Trp and Tyr followed by Förster-type energy transfer to Ret_350, Ret_400, and Ret_450.

In the second-lowest sub-figure normalized fluorescence quantum distributions are shown for $\lambda_{F,det}$ = 440 nm (contribution from Ret_400 and Ret_350), for $\lambda_{F,det}$ = 420 nm (contribution from Ret_350), and for $\lambda_{F,det}$ = 400 nm (contribution from Ret_350 and from Trp). $E'_{ex,\lambda_{F,\det}=440\ nm,\ 420\ nm,\ 400\ nm}(\lambda < 310\ nm)$ is dominated by photo-excitation of apoprotein Trp and Tyr followed by Förster-type energy transfer to Ret_350 and Ret_400.

In the bottom sub-figure normalized fluorescence quantum distributions are shown for $\lambda_{F,det}$ = 380 nm (contribution from Ret_350 and from Trp), for $\lambda_{F,det}$ = 360 nm (contribution from Ret_350 and from Trp), and for $\lambda_{F,det}$ = 340 nm (contribution from Trp).

S6. Temporal Development of Attenuation Coefficient Spectra of QuasAr1 Stored at Room Temperature

The temporal development of the attenuation coefficient spectra $\alpha(\lambda)$ of QuasAr1 in pH 8 Tris buffer in the dark at room temperature is shown in Figure S6. The attenuation decreased around 580 nm (PRSB, Ret_580), new attenuation built-up and decreased around 500 nm (PRSB, Ret_500). The attenuation increased with storage time around 400 nm (RSB, Ret_400) and around 350 nm (RSB, Ret_350). The temporal increase of attenuation in the range from 320 nm to 250 nm is attributed to apoprotein restructuring with enlarged apoprotein absorption strength.

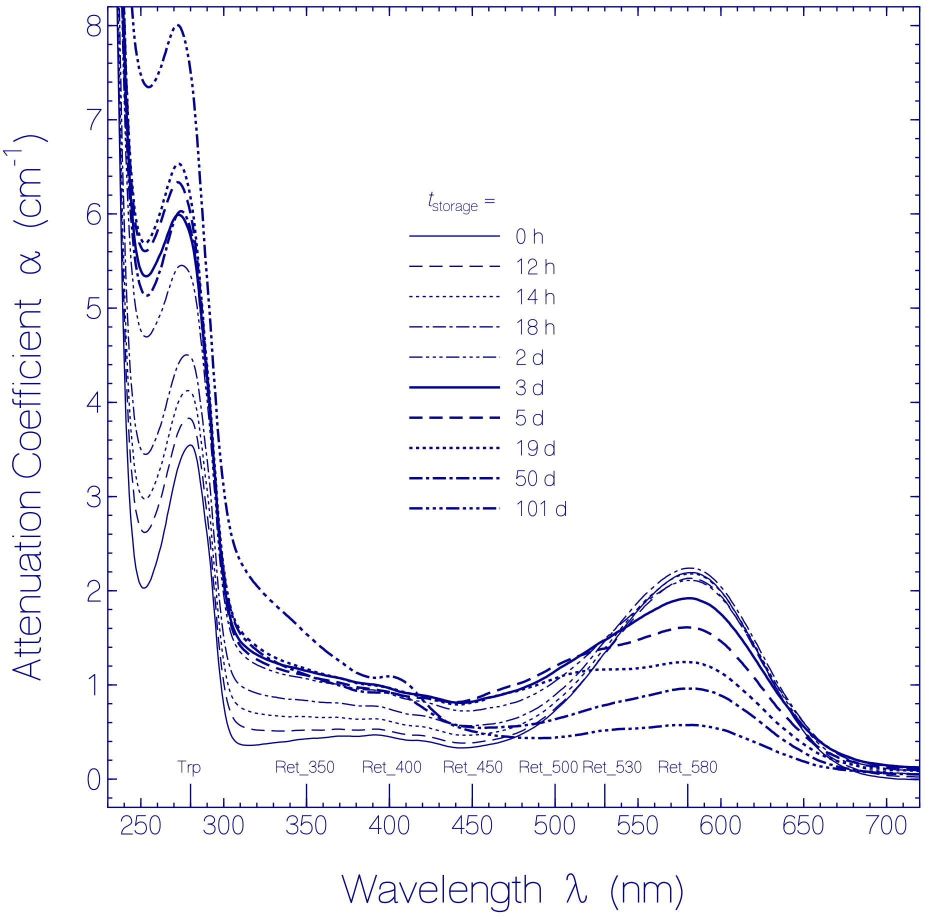

Figure S6. Temporal development of attenuation coefficient spectra $\alpha(\lambda)$ of QuasAr1 in pH 8 Tris buffer at room temperature (ϑ = 21–25 °C) in the dark. The storage times are listed in the legend.

S7. Fluorescence Emission Spectra of QuasAr1 Stored at Room Temperature for 101 Days

In Figure S7 fluorescence quantum distributions of QuasAr1 in Tris 8 buffer stored for 101 days in the dark at room temperature are displayed. The curves are similar to curves shown in Figure 8 for sample storage of 50 days at room temperature in the dark. The main difference is the enhanced overall fluorescence quantum distribution in the second top part of Figure S7 since there the absorption contribution of Ret_580 is reduced compared to the absorption contribution of Ret_530 and Ret_450 with higher specific fluorescence quantum yields.

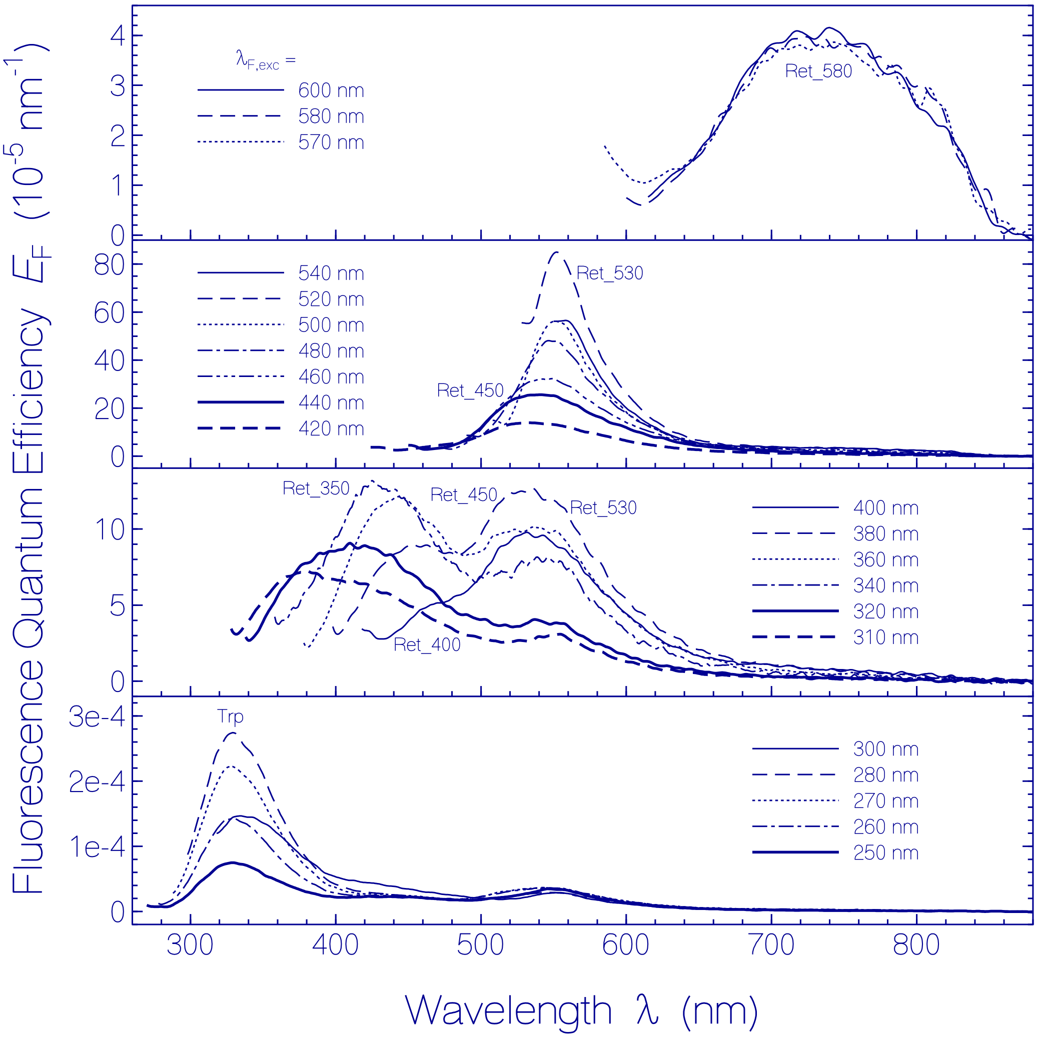

Figure S7. Fluorescence emission quantum distributions $E_F(\lambda)$ of QuasAr1 in pH 8 Tris buffer stored at room temperature (ϑ = 21–25 °C) for a duration of 101 days. The fluorescence excitation wavelengths $\lambda_{F,exc}$ are listed in the legends.

S8. Normalized Fluorescence Excitation Quantum Distributions of QuasAr1 Stored at Room Temperature

Normalized fluorescence excitation quantum distributions $E'_{ex}(\lambda) = E'_{ex,\lambda_{F,det}}(\lambda) = E_{ex,\lambda_{F,det}}(\lambda)\alpha_a(582\ nm) / E_{ex,\lambda_{F,det}=720\ nm}(\lambda = 582\ nm)$ of QuasAr1 in pH 8 Tris buffer stored in the dark at room temperature (ϑ = 21-25 °C) are shown in Figure S8 for 50 days of storage and in Figure S9 for 101 days of storage. The curves belong to different fluorescence detection wavelengths $\lambda_{F,det}$ which are listed in the legends. For comparison the absorption coefficient spectra $\alpha_a(\lambda)$ of the QuasAr1 sample stored in the dark for 50 days (Figure S8) and for 101 days (Figure S9) are included in the sub-figures.

In the top sub-figures $E'_{ex}(\lambda)$ curves for $\lambda_{F,det}$ = 780 nm, 740 nm, 700 nm, 680 nm, and 660 nm are presented. The curves belonging to $\lambda_{F,det}$ = 780 nm, 740 nm, and 700 nm resemble the absorption coefficient spectrum of Ret_580 in the wavelength range λ ≥ 310 nm. For λ < 310 nm the curves are dominated by apoprotein Trp and Tyr absorption and subsequent Förster-type energy transfer to Ret_580. The curves belonging to $\lambda_{F,det}$ = 680 nm and 660 nm include already emissions from shorter wavelength absorbing retinal isomers.

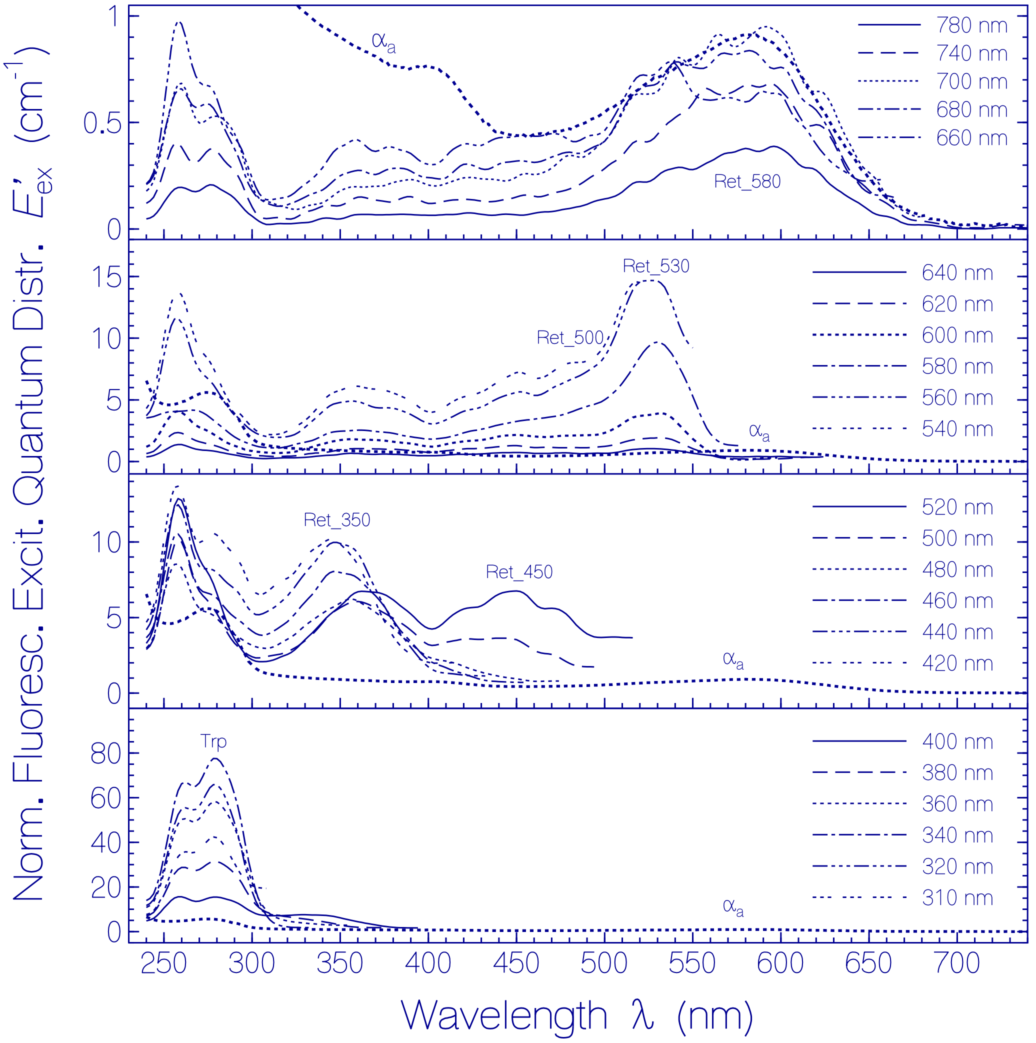

Figure S8. Normalized fluorescence excitation quantum distributions $E'_{ex,\lambda_{F,det}}(\lambda) = E_{ex,\lambda_{F,det}}(\lambda) \times \alpha_a(582\ nm) / E_{ex,\lambda_{F,det}=720\ nm}(582\ nm)$ of QuasAr1 in pH 8 Tris buffer stored in the dark at room temperature (ϑ = 21–25 °C) over a period of 50 days. The fluorescence detection wavelengths $\lambda_{F,det}$ are listed in the legends.

In the second-top sub-figures $E'_{ex}(\lambda)$ curves are displayed belonging to $\lambda_{F,det}$ = 640 nm, 620 nm, 600 nm, 580 nm, 560 nm and 540 nm. They are dominated by the emission of Ret_530. For $\lambda_{F,det}$ = 580 nm, $\lambda_{F,det}$ = 560 nm and 540 nm contributions are present from Ret_450, Ret_400 and Ret_350. Below λ = 300 nm the curves are dominated by apoprotein Trp and Tyr absorption and subsequent Förster-type energy transfer to Ret_350, Ret_400, Ret_450, and Ret_530.

In the second-lowest sub-figures normalized fluorescence excitation quantum distributions belonging to $\lambda_{F,det}$ = 520 nm and 500 nm are determined by emission from Ret_450, Ret_400, Ret_350 and Förster-type energy transfer from apoprotein Trp and Tyr to Ret_350, Ret_400 and Ret_450. The curves belonging to $\lambda_{F,det}$ = 480 nm, 460 nm, 440 nm and 420 nm are determined by emissions from Ret_400, Ret_350, and Förster-type energy transfer from Trp and Tyr to Ret_400 and Ret_350.

In the bottom sub-figures the normalized fluorescence excitation quantum distributions belonging to $\lambda_{F,det}$ = 400 nm and 380 nm indicate fluorescence emission from Ret_350. The curves belonging to $\lambda_{F,det}$ = 360 nm, 340 nm, 320 nm and 310 nm are determined by emission from Trp and Förster-type energy transfer from Tyr to Trp.

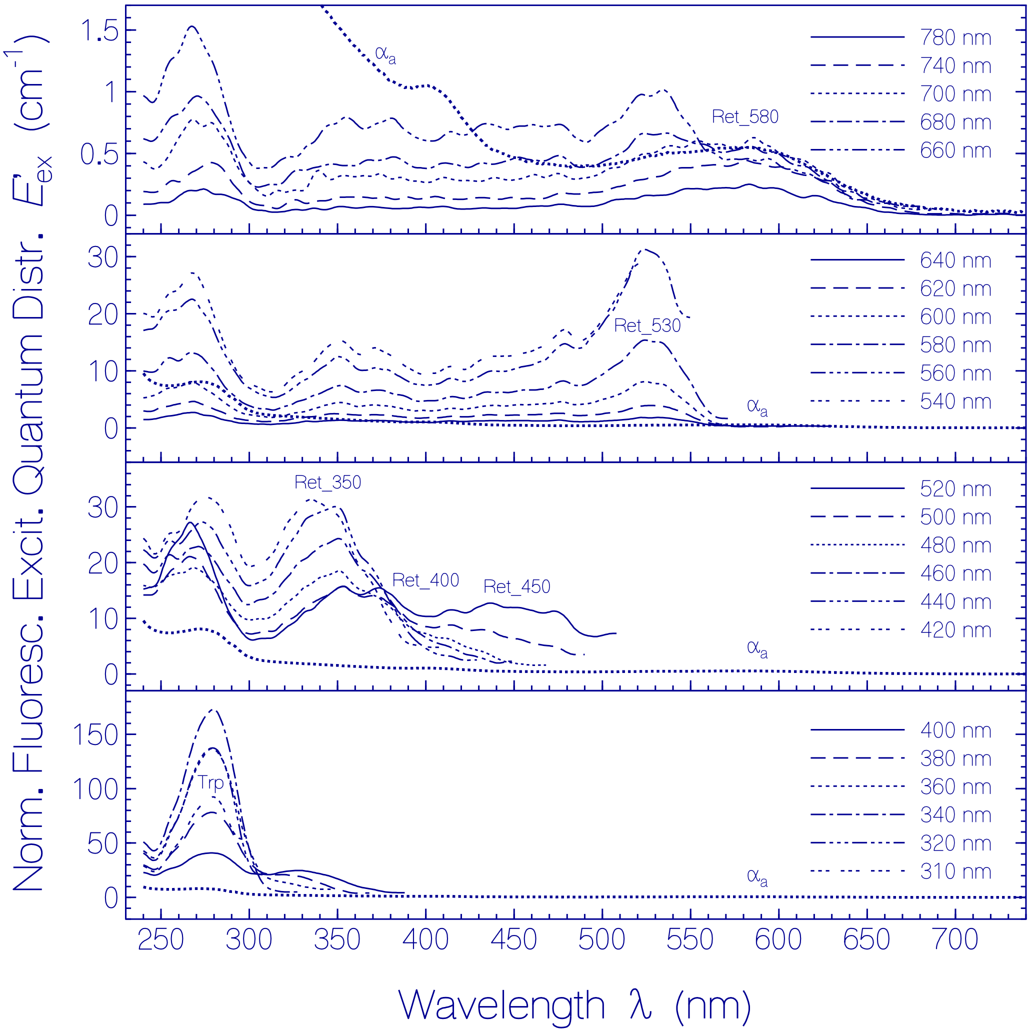

Figure S9. Normalized fluorescence excitation quantum distributions $E'_{ex,\lambda_{F,det}}(\lambda) = E_{ex,\lambda_{F,det}}(\lambda) \times \alpha_a(582\ nm) / E_{ex,\lambda_{F,det}=720\ nm}(582\ nm)$ of QuasAr1 in pH 8 Tris buffer stored in the dark at room temperature (ϑ = 21–25 °C) over a period of 101 days. The fluorescence detection wavelengths $\lambda_{F,det}$ are listed in the legends.

S9. Primary Photo-isomerization Behavior of Some Rhodopsins

In Table S1 absorption, fluorescence, and first-step photo-isomerization parameters of some microbial rhodopsins (type I rhodopsins) and of an animal rhodopsin (type II rhodopsin) are listed.

Table S1. Absorption, fluorescence, and primary photo-isomerization comparison of rhodopsins.

Name	Buffer	Cofactor	$\lambda_{a,max}$ (nm)	$\lambda_{F,max}$ (nm)	$\delta\tilde{\nu}_{Stokes}$ (cm^{-1})	ϕ_F	τ_F (ps)	Photo-isomer	Reference
BRh	pH 6.8–7	$PRSB_{11\text{-}cis}$	498	640 [40]	4455	6×10^{-4} [86]<br>1.2×10^{-5} [87]<br>9×10^{-6} [40]	0.125–0.33, 1.0–2.4	$PRSB_{all\text{-}trans}$	[88]
PR	pH 8	$PRSB_{all\text{-}trans}$	521	700	4900	2.6×10^{-4}	0.45, 4	$PRSB_{13\text{-}cis}$	[41]
BR	pH 7.5	$PRSB_{all\text{-}trans}$	568	755 ± 10 [89]	≈ 4360	1×10^{-4} [90]<br>1.3×10^{-4} [91]	< 0.15, ≈ 0.45<br>0.2, 0.5 [92]<br>≈ 0.2, ≈ 0.7,≈ 10 [93]	$PRSB_{13\text{-}cis}$	[94]
HR	pH 7	$PRSB_{all\text{-}trans}$	578	≈ 685	≈ 2700	5×10^{-4}	5<br>1.5, 6.6 [95]<br>1.5, 8.5 [96]	$PRSB_{13\text{-}cis}$	[97]
sSRI	pH 6	$PRSB_{all\text{-}trans}$	587	≈ 700	≈ 2570	1.4×10^{-3}	5, 33	$PRSB_{13\text{-}cis}$	[91]
sSRII	pH 6	$PRSB_{all\text{-}trans}$	487				< 0.15, 0.4, 4	$PRSB_{13\text{-}cis}$	[91]
pSRII	pH 8	$PRSB_{all\text{-}trans}$	497	630	4250	1.2×10^{-4}	0.08–0.25, 1.7–2.5 [98]	$PRSB_{13\text{-}cis}$	[91]
ChR-2	pH 7.4	$PRSB_{all\text{-}trans}$	475	530	2185		0.4	$PRSB_{13\text{-}cis}$	[99]
ASR	pH 7.5	$PRSB_{all\text{-}trans}$	549				≈ 0.15	$PRSB_{13\text{-}cis}$	[100]
ASR	pH 7.5	$PRSB_{13\text{-}cis}$	537				≈ 0.72	$PRSB_{all\text{-}trans}$	[100]
HKR1	pH 7.4	$RSB_{13\text{-}cis}$	380	≈ 430	≈ 3060	1.5×10^{-3}	5, 60 [101]	$RSB_{all\text{-}trans}$	[56]
HKR1	pH 7.4	P $RSB_{all\text{-}trans}$	485	≈ 530	≈ 1750	2.8×10^{-4}	0.65, 5 [102]	$PRSB_{13\text{-}cis}$	[56]
BeRh	pH 8	$PRSB_{all\text{-}trans}$	530	≈ 640	≈ 3250	1.7×10^{-4}	≈ 1.2	$PRSB_{13\text{-}cis}$	[60]
CaRh	pH 7.3	$PRSB_{all\text{-}trans}$	540	≈ 690	≈ 4030	1.1×10^{-5}	0.088	$PRSB_{13\text{-}cis}$	103]
Arch	pH 7.3	$PRSB_{all\text{-}trans}$ [S20]	558	687	3365	9×10^{-4}		$PRSB_{13\text{-}cis}$ [S20]	[36]
QuasAr1	pH 8	PRSB	580	≈ 740	≈ 3730	6.5×10^{-3}	≈ 61.5	PRSB	This work

Abbreviations: BRh: bovine rhodopsin (type-II rhodopsin). PR: proteorhodopsin from uncultivated marine γ-proteobacteria. BR: bacteriorhodopsin from *Halobacterium salinarum*. HR: halorhodopsin from *Halobacterium halobium*. sSRI: sensory rhodopsin I from *Halobacterium salinarum*. sSRII: sensory rhodopsin II from

Halobacterium salinarum. pSRII: sensory rhodopsin II from *Natronobacterium pharaonis*. ChR-2: channelrhodopsin 2 from *Chlamydomonas reinhardtii*. ASR: sensory rhodopsin from *Anabaena* (*Nosto*c) sp. PCC 7120 cyanobacterium. HKR1: histidine kinase rhodopsin 1 from *Chlamydomonas reinhardtii*. BeRh: rhodopsin domain of the rhodopsin-guanylyl cyclase from *Blastocladiella emersonii*. CaRh: rhodopsin domain of the rhodopsin-guanylyl cyclase from *Catenaria anguillulae.* Arch: Archaerhodopsin 3 from *Halorubrum sodomense*. QuasAr1: fluorescent voltage sensor 1 of quality superior to Arch from *Halorubrum sodomense*. PRSB: protonated retinal Schiff base. RSB: deprotonated retinal Schiff base. $\lambda_{a,max}$: wavelength position of maximum absorption of first absorption band. $\lambda_{F,max}$: wavelength position of maximum fluorescence. ϕ_F: fluorescence quantum yield. τ_F: fluorescence lifetime. $\delta\tilde{\nu}_{Stokes} = \lambda_{a,\max}^{-1} - \lambda_{F,\max}^{-1}$: Stokes shift = the wavenumber difference between the maximum of the absorption band and the maximum of the corresponding fluorescence band.
